# Mutation of *RGG2*, which encodes a type B heterotrimeric G protein γ subunit, increases grain size and yield production in rice

**DOI:** 10.1111/pbi.13005

**Published:** 2018-12-13

**Authors:** Jun Miao, Zefeng Yang, Dongping Zhang, Yuzhu Wang, Mengbin Xu, Lihui Zhou, Jun Wang, Shujun Wu, Youli Yao, Xi Du, Fangfei Gu, Zhiyun Gong, Minghong Gu, Guohua Liang, Yong Zhou

**Affiliations:** ^1^ Jiangsu Key Laboratory of Crop Genetics and Physiology/Co‐Innovation Center for Modern Production Technology of Grain Crops Key Laboratory of Plant Functional Genomics of the Ministry of Education Yangzhou University Yangzhou China; ^2^ Institute of Food Crops Jiangsu Academy of Agricultural Sciences Nanjing China; ^3^ Shanghai Academy of Agricultural Sciences Shanghai China

**Keywords:** heterotrimeric G protein, RGG2, rice, grain size, yield production

## Abstract

Heterotrimeric G proteins, which consist of G_α_, G_β_ and G_γ_ subunits, function as molecular switches that regulate a wide range of developmental processes in plants. In this study, we characterised the function of rice *RGG2*, which encodes a type B G_γ_ subunit, in regulating grain size and yield production. The expression levels of *RGG2* were significantly higher than those of other rice G_γ_‐encoding genes in all tissues tested, suggesting that *RGG2* plays essential roles in rice growth and development. By regulating cell expansion, overexpression of *RGG2* in Nipponbare (NIP) led to reduced plant height and decreased grain size. By contrast, two mutants generated by the clustered, regularly interspaced, short palindromic repeat (CRISPR)/CRISPR‐associated protein 9 (Cas9) system in the Zhenshan 97 (ZS97) background, *zrgg2‐1* and *zrgg2‐2*, exhibited enhanced growth, including elongated internodes, increased 1000‐grain weight and plant biomass and enhanced grain yield per plant (+11.8% and 16.0%, respectively). These results demonstrate that *RGG2* acts as a negative regulator of plant growth and organ size in rice. By measuring the length of the second leaf sheath after gibberellin (GA_3_) treatment and the GA‐induced α‐amylase activity of seeds, we found that *RGG2* is also involved in GA signalling. In summary, we propose that *RGG2* may regulate grain and organ size via the GA pathway and that manipulation of *RGG2* may provide a novel strategy for rice grain yield enhancement.

## Introduction

Heterotrimeric G proteins are versatile components of the transmembrane signal transduction pathways associated with a wide range of growth and developmental responses in plants, fungi and animals. The classic heterotrimers include three different subunits, α, β and γ, which are organized into a highly conserved structure. After being activated by an external stimulus, these heterotrimers are directly regulated by G protein‐coupled receptors (GPCRs). In humans, at least 23 G_α_, 5 G_β_ and 12 G_γ_ subunits have been identified, and they allow for a large number of different heterotrimer combinations (McCudden *et al*., 2005; McIntire *et al*., 2002; Robishaw and Berlot, 2004).

Plant G proteins are also involved in a series of signalling processes, such as hormone responses (Jin *et al*., 2013; Okamoto *et al*., 2009; Pandey and Assmann, 2004; Wang *et al*., 2006), plant defence responses (Cheng *et al*., 2015; Liang *et al*., 2016; Liu *et al*., 2013), plant shoot meristem development (Bommert *et al*., 2013; Ishida *et al*., 2014), abiotic stress responses (Shi *et al*., 2015; Yu and Assmann, 2015) and light perception (Botto *et al*., 2009; Jones *et al*., 2003). In contrast to complicated mammalian systems, most plants have only one G_α_, one G_β_ and several G_γ_ subunits. For example, the *Arabidopsis thaliana* genome includes one canonical G protein α subunit (GPA1), one G protein β subunit (AGB1), and at least three G protein γ subunit (AGG1, AGG2 and AGG3) (Temple and Jones, 2007). The loss‐of‐function of Arabidopsis *GPA1* or *AGB1* leads to defects in plant growth and development, whereas mutations in *AGG1* or *AGG2* have little effect on organ growth (Trusov *et al*., 2007, 2008; Ullah *et al*., 2001). AGG3 was the last identified G_γ_ subunit in Arabidopsis, and it has been reported to affect guard cell K^+^ channels, morphological development and abscisic acid (ABA) responses (Chakravorty *et al*., 2011). Constitutive expression of *AGG3* increases organ size in Arabidopsis (Li *et al*., 2012), enhances oil production in *Camelina sativa* (Roy Choudhury *et al*., 2014) and promotes yield and stress responses in *Setaria viridis* (Kaur *et al*., 2018). The phenotype of the Arabidopsis G_γ_ triple mutant (*agg1agg2agg3*) mimics that of *agb1*, suggesting that all the members of the G protein family have been discovered in Arabidopsis (Thung *et al*., 2012).

In the rice genome, one G_α_ gene (*RGA1*), one G_β_ gene (*RGB1*) and five G_γ_ homologoues genes (*RGG1*,* RGG2*,* GS3*,* qPE9‐1*/*DEP1* and *GGC2*) have been identified (Sun *et al*., 2018). *RGA1* has been reported to be involved in gibberellin (GA) signal transduction and brassinosteroid responses, and the *RGA1‐*deficient mutant *d1* shows a dwarf and small‐seed phenotype (Ashikari *et al*., 1999; Fujisawa *et al*., 1999). *RGA1* has also been shown to play essential roles in drought tolerance, photoprotection and photoavoidance in rice (Ferrero‐Serrano and Assmann, 2016; Ferrero‐Serrano *et al*., 2018). Although *RGB1* mutants have not been isolated in rice, the suppression of *RGB1* causes growth abnormalities, such as dwarfism and short grain length (Utsunomiya *et al*., 2011). *GS3* has been identified as a major QTL for grain length and weight, and the *gs3* allele leads to larger and heavier grains (Fan *et al*., 2006; Mao *et al*., 2010; Takano‐Kai *et al*., 2009). In rice, multiple roles have been identified for *qPE9‐1/DEP1*, including the regulation of grain size, panicle development and responses to nitrogen (Huang *et al*., 2009; Sun *et al*., 2014; Zhou *et al*., 2009). We further found that *qPE9‐1/DEP1* is modulated by *RGB1* and functions as a negative player in ABA‐dependent drought stress responses (Zhang *et al*., 2015), which highlights the complexity of G protein components in rice. Recently, the *RGG1* gene was reported to increase salinity stress tolerance in rice by elevating detoxification of reactive oxygen species (Swain *et al*., 2017).

However, the biological function of the *RGG2* gene in rice has not been characterized. In this study, we provide evidence that RGG2 and RGB1 interact together as a complex and that *RGG2* plays multiple roles in rice plant architecture, grain size and yield production.

## Results

### RGG2 is a type B G_γ_ subunit and interacts with RGB1

**Figure 1:**
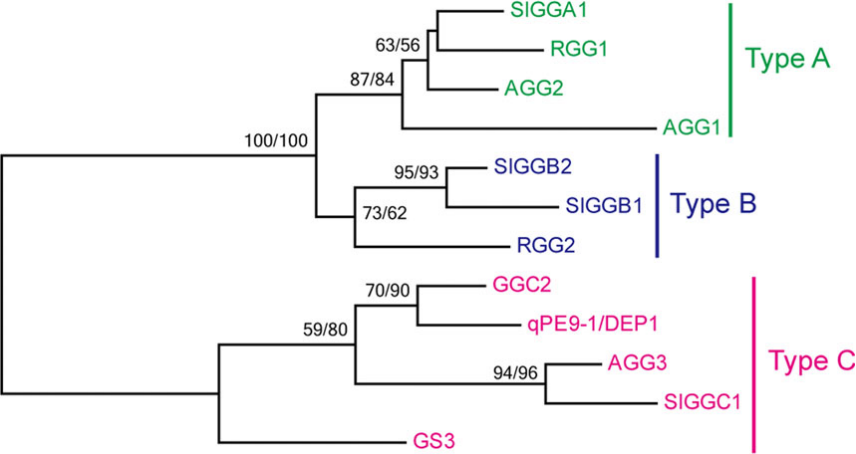
Phylogenetic tree of G_γ_ subunits from rice, Arabidopsis and tomato.

Phylogenetic analysis revealed that rice RGG1 is a type A G_γ_ protein similar to the Arabidopsis AGG1 and AGG2 proteins and the tomato SlGGA1 protein (Figure 1). Type A G_γ_ subunits represent canonical G proteins that contain relatively few amino acid residues and a CaaX motif at their C‐terminal end (Figure S1), and these subunits are structurally similar to their counterparts in animals. RGG2 is a member of the type B G_γ_ proteins, which show similarity to the type A G_γ_ subunits. However, the type B family lack the C‐terminal CaaX motif. RGG2 has a SDFS motif in the C‐terminal region (Figure S1), which is similar to the type B G_γ_ proteins in other monocot species (Trusov *et al*., 2012). However, type B G_γ_ proteins have not been identified in the Arabidopsis genome. Because of their large protein size and long cysteine‐rich C‐terminal end, the rice GS3, qPE9‐1/DEP1 and GGC2 proteins, Arabidopsis AGG3 protein and tomato SlGGC1 protein form a type C subgroup (Figure 1). The similarity percentage between the type A and B G_γ_ proteins and the type C proteins is relatively low (Table S1), and most of the similarities are limited to the conserved GGL (G gamma‐like) domain (Figure S1).

**Figure 2:**
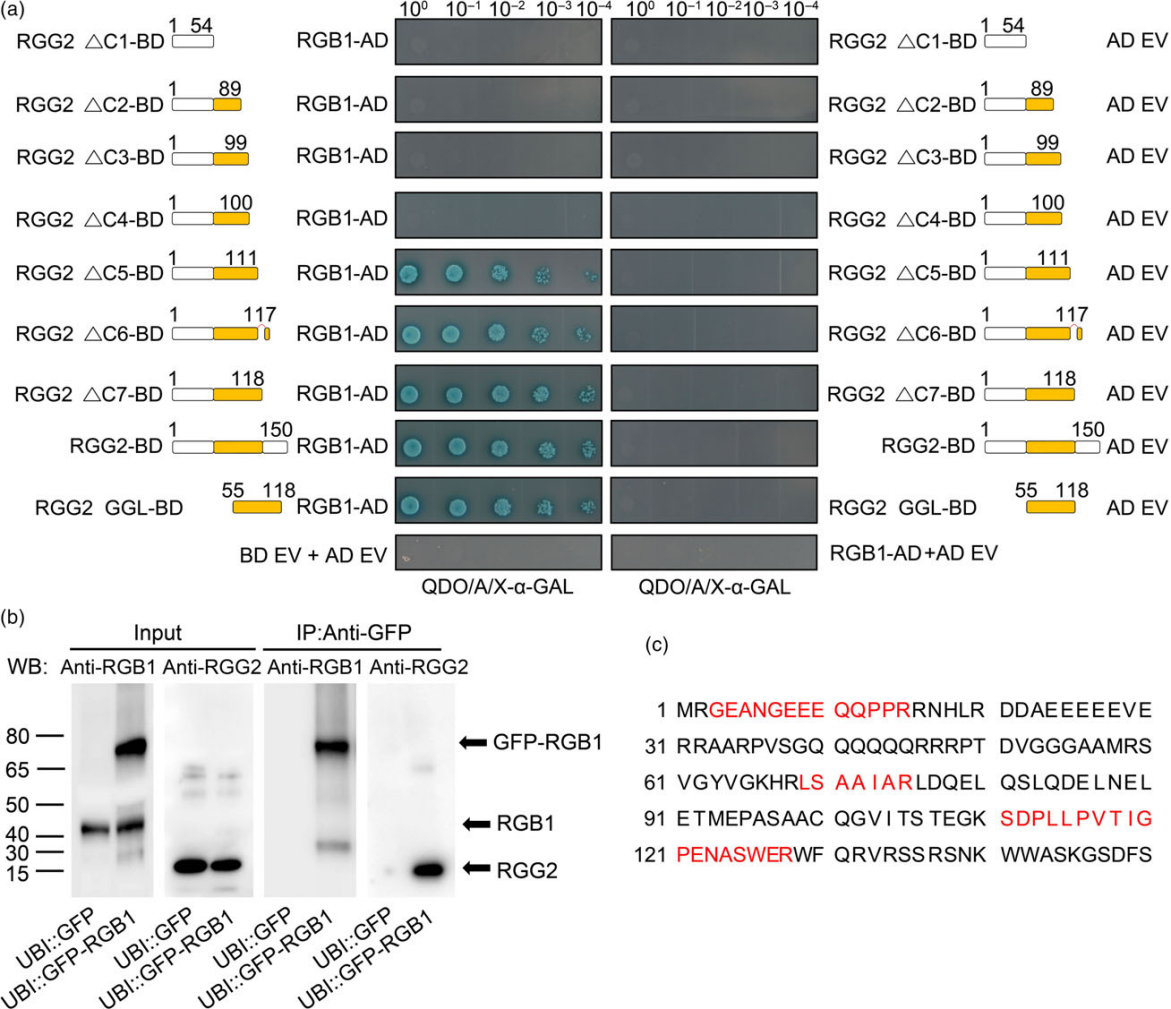
Protein‐protein interactions between RGG2 and RGB1. (a) Yeast two‐hybrid analysis. Schematic representation of the truncated RGG2 proteins used for the yeast two‐hybrid assay. In the assay, RGG2 is used as bait (GAL4‐BD, BD) and RGB1 is used as prey (GAL4‐AD, AD). ΔC shows the C‐terminally truncated RGG2 deletion variants. The numbers represent the different lengths of each truncated RGG2 protein. GGL is the G gamma‐like domain, and DPLP is the conserved motif within the C‐terminus of RGG2. QDO/A/X‐α‐GAL represents SD/‐Leu‐Trp‐His‐Ade+X‐α‐GAL medium. (b) Co‐immunoprecipitation assays *in vivo*. Total protein was extracted from transgenic plants expressing GFP‐tagged RGB1, and plants expressing GFP were used as a negative control. Proteins were immunoprecipitated with anti‐GFP magnetic beads, and the precipitated proteins were detected using both RGB1 and RGG2 antibodies. (c) Analysis of the immunoprecipitated proteins by LC‐MS/MS assays. The identified RGG2 peptides are shown with red.

Using a yeast two‐hybrid system, we confirmed that RGG2 and RGB1 interact together (Figure 2a). To further investigate the interaction between different domains of RGG2 and RGB1, several truncated RGG2 proteins were generated. As shown in Figure 2a, the GGL domain of RGG2 (amino acids 55–118) can strongly interact with RGB1 and the conserved DPLP motif in the C‐terminal is not essential for G_βγ_ interaction. The interaction between RGG2 and RGB1 was also confirmed by co‐immunoprecipitation assays *in vivo*. Total proteins were extracted from UBI::GFP‐RGB1 and UBI::GFP transgenic rice seedlings, and all immunoprecipitated proteins were eluted from anti‐GFP magnetic beads. The RGG2 protein was detected in the immunoprecipitated proteins by using anti‐RGG2 antibody (Figure 2b). Moreover, immunoprecipitated proteins were subjected to Liquid Chromatography‐Mass Spectrometry/Mass Spectrometry (LC‐MS/MS) to analyze the RGB1 interacting proteins. Three peptides that accounted for 25.3% of the total RGG2 protein presented an interaction between RGG2 and RGB1 in UBI::GFP‐RGB1 transgenic rice (Figure 2c), whereas the RGG2 protein was not found in UBI::GFP transgenic rice. These data indicate that RGG2 interacts with RGB1.

### Expression pattern and subcellular localization

**Figure 3:**
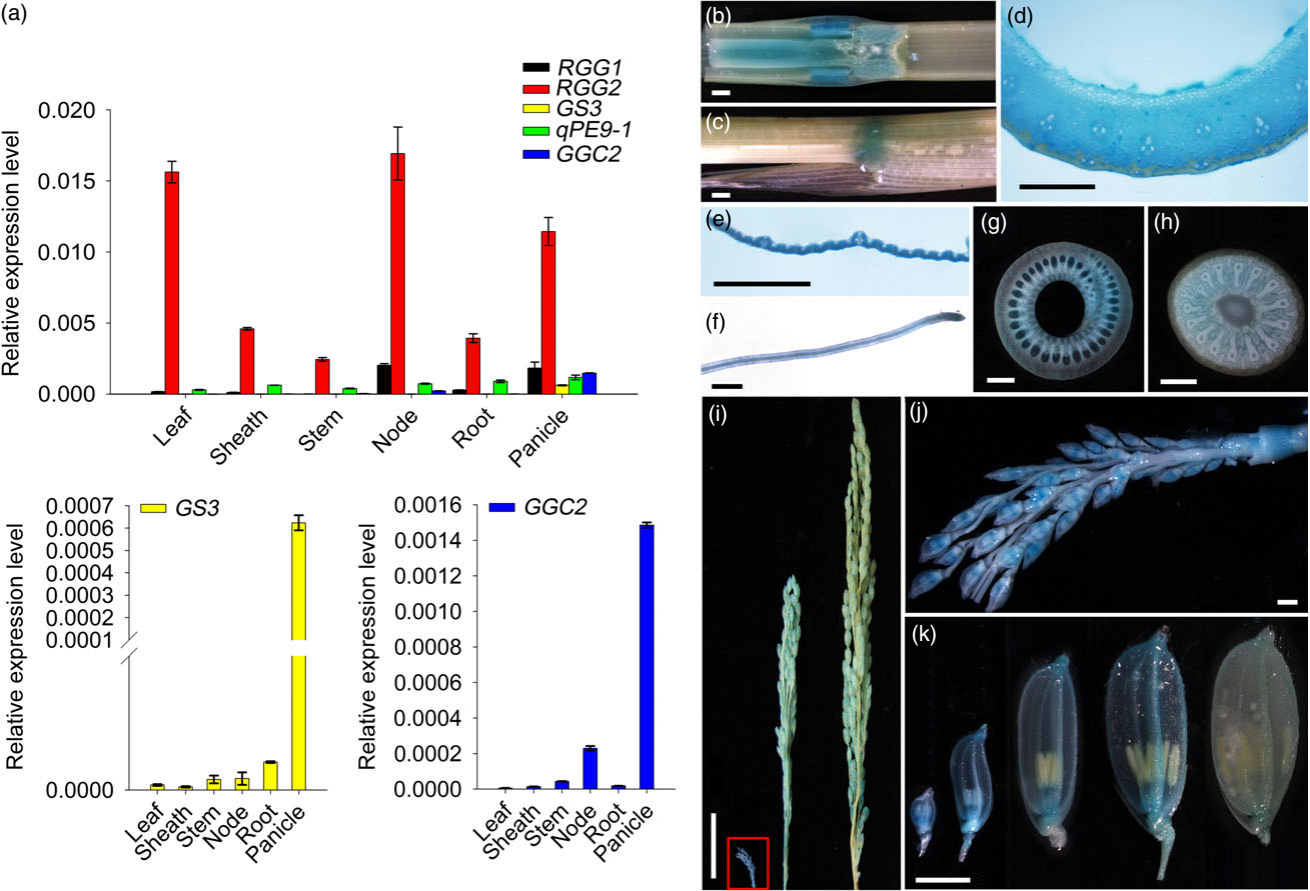
Expression pattern of *RGG2*. (a) Transcript levels of five rice G_γ_ subunit‐encoding genes in various tissues detected by qPCR. *OsActin* was used as the internal control. The magnified images of *GS3* and *GGC2* expression pattern are further displayed at the bottom of the figure because the expression levels of these two genes are extremely low. (b) GUS activity in a stem and node longitudinal section. Bar = 1 mm. (c) GUS activity in a sheath. Bar = 1 mm. (d) GUS activity in a stem cross‐section. Bar = 1 mm. (e) GUS activity in a leaf. Bar = 1 mm. (f) GUS activity in a root. Bar = 1 mm. (g) GUS activity in a sheath cross‐section. Bar = 1 mm. (h) GUS activity in a node cross‐section. Bar = 1 mm. (i) GUS activity in panicles at different growth stages. Bar = 3 cm. (j) A magnified image of the boxed young inflorescence shown in (i). Bar = 1 mm. (k) GUS activity in spikelet hulls from 2‐ to 3‐, 8‐ to 9‐, 12‐ to 13‐, 19‐ to 20‐ and 23‐ to 24‐cm‐long inflorescences. Bar = 2 mm.

Quantitative reverse transcription‐PCR (qPCR) analysis revealed that *RGG2* was constitutively expressed in all plant tissues, including the leaf, sheath, stem, panicle, node and root (Figure 3a). Using transgenic plants expressing the β‐glucuronidase (GUS) reporter gene under the control of its native promoter, we further analyzed the expression pattern of *RGG2*. Strong GUS staining was detected in various tissues (Figure 3b–k). We also observed that the GUS activities in the spikelet hulls from developed inflorescences were much lower than those from young inflorescences (Figure 3i–k), which was consistent with the expression levels of *RGG2* in the panicles at different developmental stages (Figure S2a). These data suggested that the transcript accumulation of *RGG2* decreased as the inflorescence and grain development. The expression levels of the other four rice G_γ_‐encoding genes were quantified. Surprisingly, the five G_γ_ genes in rice displayed distinct expression abundances (Figure 3a). A notably higher transcript accumulation of *RGG2* was detected in both vegetative and reproductive tissues where the expression levels of *RGG1*,* GS3*,* qPE9‐1*/*DEP1* and *GGC2* were low. These data suggest that *RGG2* is the most abundantly expressed G_γ_ gene in rice.

**Figure 4:**
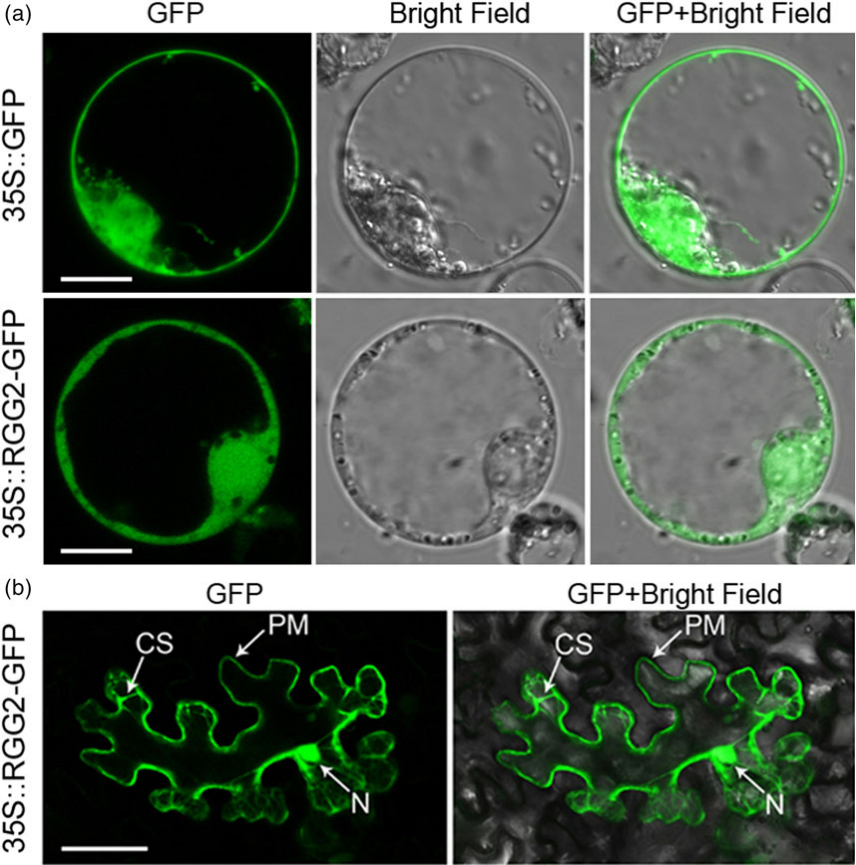
Subcellular localization of the RGG2 protein. (a) 35S::GFP and 35S::RGG2‐GFP in rice protoplasts. Bars = 10 μm. (b) 35S::RGG2‐GFP in *N. benthamiana* cells. Bar = 100 μm. CS, cytoplasm; PM, plasma membrane; and N, nucleus.

To determine the subcellular localization of RGG2, we transiently expressed both green fluorescent protein (GFP) alone and an RGG2‐GFP fusion protein under the control of the CaMV 35S promoter in rice protoplasts. Similar to the free GFP localization pattern, the green fluorescence pattern of RGG2‐GFP exhibited ubiquitous distribution in the nucleus, cytoplasm and plasma membrane (Figure 4a). A similar result was also observed in *Nicotiana benthamiana* leaves expressing the RGG2‐GFP fusion protein (Figure 4b). These data indicate that the RGG2 subunit is localized to the nucleus, cytoplasm and plasma membrane.

### Overexpression of *RGG2* causes dwarfism and small grains

To decipher the functional role of *RGG2* in plant development, we created an overexpression construct. We then used *Agrobacterium tumefaciens*‐mediated transformation to transform the construct into Nipponbare (NIP) and produced more than 20 independent transgenic rice lines. The insertion and overexpression of *RGG2* were confirmed by genomic DNA PCR and qPCR analyses using T_0_ plants (data not shown). Two of the derived independent homozygous transgenic lines, OE1 and OE2, were chosen for further analysis.

**Figure 5:**
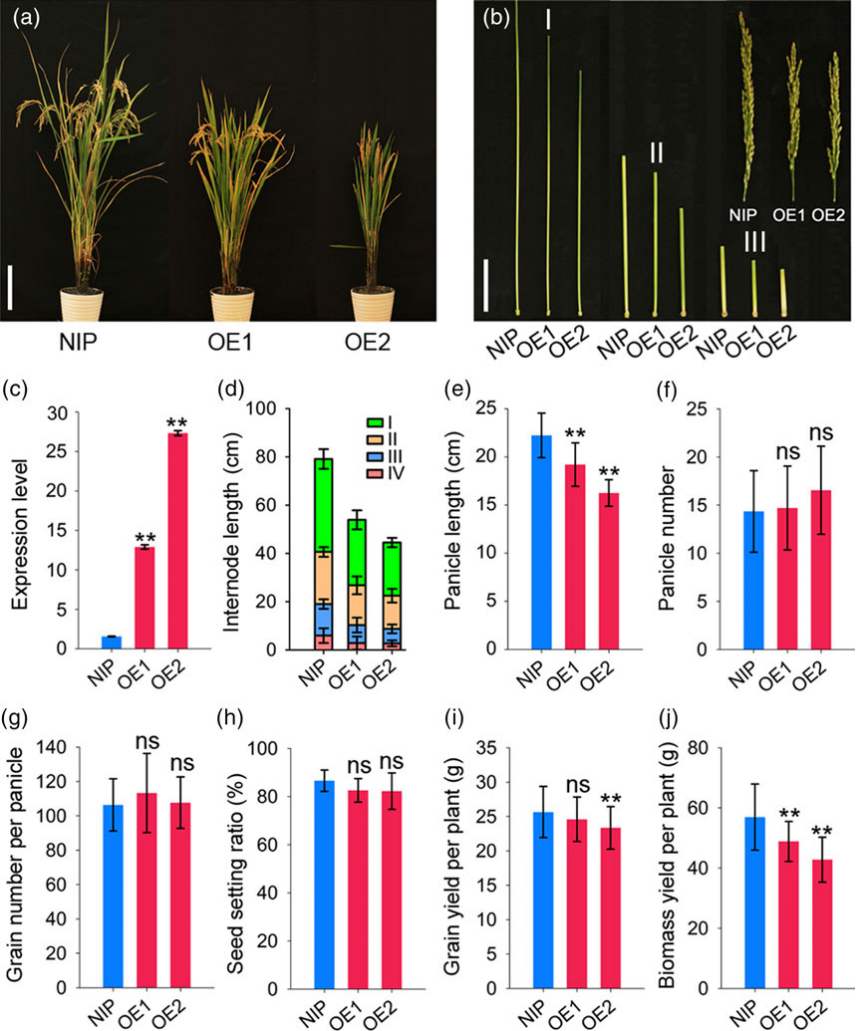
Overexpression of *RGG2* affects multiple agronomic traits. (a) Plant phenotype of NIP, OE1, and OE2 at the mature stage. Bar = 20 cm. (b) Panicles and internodes of NIP, OE1, and OE2 at the mature stage. Bar = 5 cm. (c) *RGG2* expression levels in NIP, OE1, and OE2. (d–j) Comparisons between NIP, OE1, and OE2 with respect to (d) internode length; (e) panicle length; (f) panicle number per plant; (g) grain number per panicle; (h) seed setting ratio; (i) grain yield per plant; and (j) biomass yield per plant. The data are given as the means ± SD (*n* ≥ 20). Student's *t‐*test: *P <* *0.05; **P <* *0.01; ns, not significant. I–IV represent the first to the fourth internodes from the panicles.

**Figure 6:**
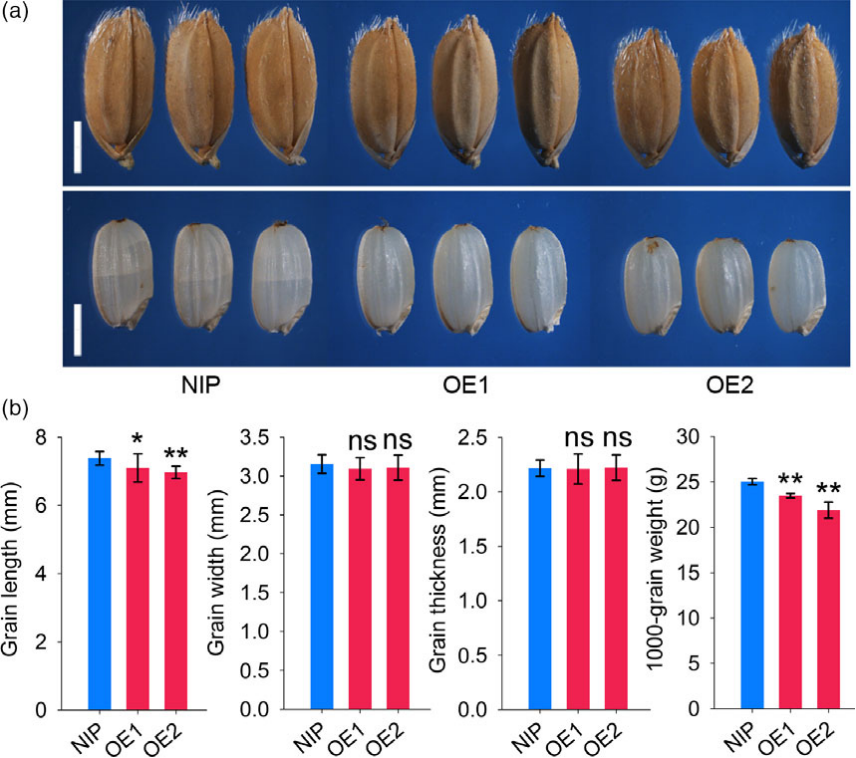
Grain performance of NIP, OE1, and OE2. (a) Grains and brown rice of NIP, OE1, and OE2. Bars = 2 mm; (b) Comparisons of grain length, width, thickness and weight among NIP, OE1 and OE2. The data are given as the means ± SD (*n* ≥ 20). Student's *t‐*test: *P <* *0.05; **P <* *0.01; ns, not significant.

The expression levels of *RGG2* in OE1 and OE2 increased by 8.3 and 17.5 fold compared to that in NIP, respectively (Figure 5c). As a result, the OE1 and OE2 transgenic plants were semi‐dwarfed and exhibited a compact plant architecture (Figure 5a). At the mature stage, the plant heights of OE1 and OE2 were only 69.3% and 61.1% that of NIP, respectively (Figure 5a). Compared to the internodes of the wild‐type lines, every internode of the overexpression lines was shortened (Figure 5b,d). Moreover, NIP produced droopy panicles while OE1 and OE2 produced short and erect panicles (Figure 5a,b,e). In addition to the compact plant architecture, the transgenic lines also produced small and round rice grains (Figure 6). The 1000‐grain weights of OE1 and OE2 decreased by 6.2% and 12.5% compared to that of NIP, respectively (Figure 6). However, other yield components, such as the panicle number, grain number per panicle and seed setting ratio, did not differ between the NIP and transgenic lines (Figure 5f–h). The grain yield per plant of OE2 significantly decreased by 9.0% and the yield production of OE1 was slightly reduced by 4.1% (*P*‐value = 0.37) compared to that of NIP (Figure 5i). We also found those the biomass yields in the transgenic lines were lower than those in wild‐type lines (Figure 5j). Taken together, these results indicate that overexpression of *RGG2* alters the plant architecture and negatively influences the grain size and yield production in rice.

### Mutation of *RGG2* increases grain size and yield production in Zhenshan 97 (ZS97)

**Figure 7:**
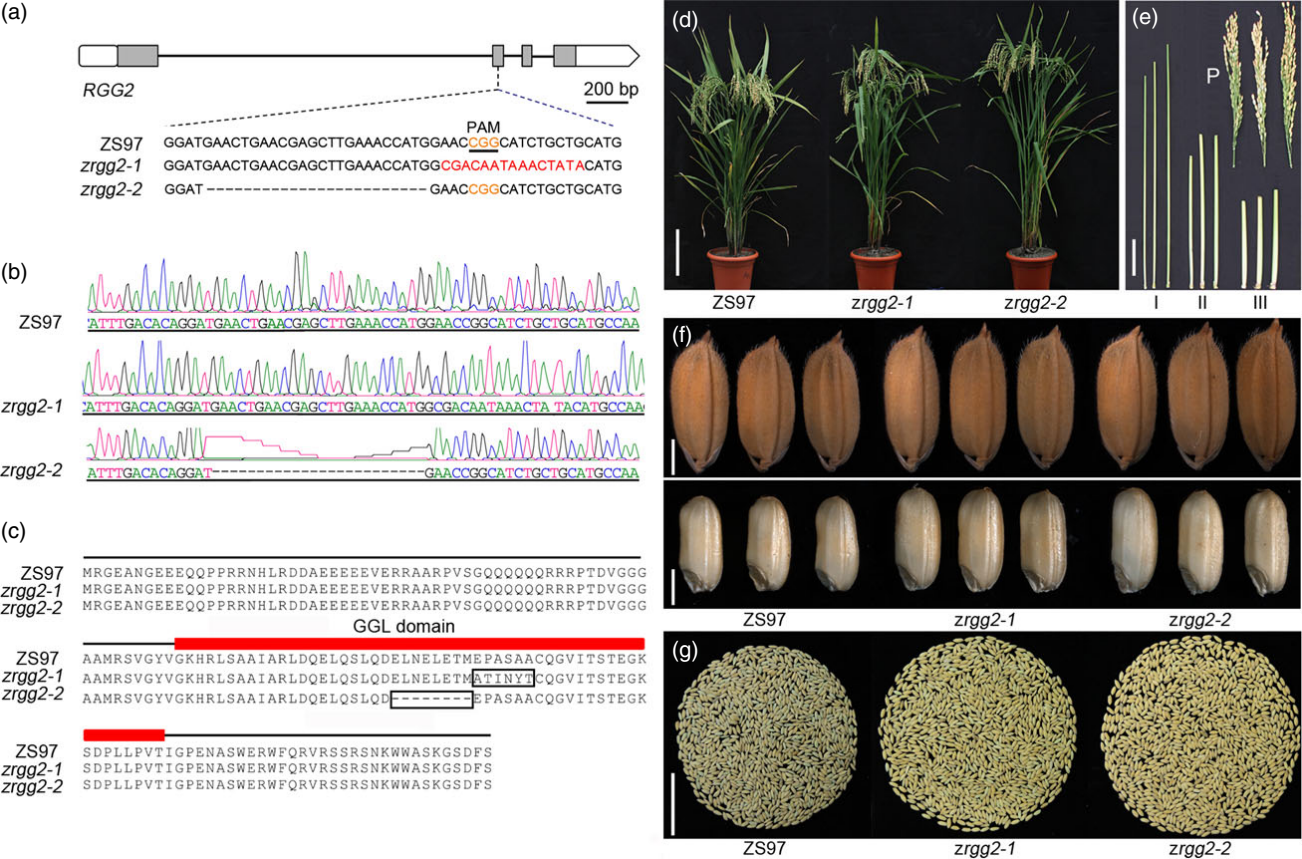
Targeted mutation of *RGG2* led to improved yield performance in Zhenshan 97 (ZS97). (a) Sequence (5ʹ‐CGAGCTTGAAACCATGGAAC‐3ʹ) located in the second exon of the *RGG2* gene was selected as the target site of sgRNA. Two types of mutation events were generated by CRISPR/Cas9 in ZS97. (b) Mutation events were confirmed by sequencing. (c) Black boxes show amino acid mutations of the RGG2 protein in the two mutants (*zrgg2‐1* and *zrgg2‐2*), and red rectangles indicate the GGL domain. (d) Comparison of the wild‐type plant and two *rgg2* mutant plants. Bar = 20 cm. (e) Comparison of the panicles and internodes between the wild‐type and two *rgg2* mutants. P indicates panicles and I–III represent the first to third internodes from the panicles. ZS97, *zrgg2‐1* and *zrgg2‐2* are shown from left to right. Bar = 5 cm. (f) Grains and brown rice of ZS97, *zrgg2‐1* and *zrgg2‐2* plants. Bars = 2 mm. (g) Grains from the whole plants of ZS97, *zrgg2‐1* and *zrgg2‐2*. Bar = 5 cm.

To further investigate the function of *RGG2*, a clustered regularly interspaced short palindromic repeats (CRISPR)/CRISPR‐associated protein 9 (Cas9) system was used to generate mutants of the *RGG2* gene. A CRISPR/Cas9 construct expressing single guide RNA (sgRNA) that targeted the GGL coding region in the second exon of *RGG2* was generated and used to transform NIP and ZS97 (Figure 7). Sequencing of PCR‐amplified *RGG2* genomic DNA from the transgenic plants revealed that one homozygous mutant of NIP (*nrgg2‐1*) and two homozygous mutants of ZS97 (*zrgg2‐1* and *zrgg2‐2*) were obtained. The *nrgg2‐1* mutant had a 6‐bp deletion at its target site, which caused a 3‐amino acid mutation (Figure S3). The mutations in *zrgg2‐1* and *zrgg2‐2* yielded a 15‐bp substitution and a 24‐bp deletion, respectively (Figure 7a,b) and were predicted to produce a 6‐amino acid substitution and an 8‐amino acid deletion, respectively (Figure 7c).

The *nrgg2‐1*,* zrgg2‐1*,* zrgg2‐2* and wild‐type plants were grown in experimental plots in 2017 and used for detailed phenotypic analysis. As shown in Figure S4 and Table S2, a slight but significant increase in plant height and panicle length was observed in *nrgg2‐1*. There were very small changes in grain size and weight between *nrgg2‐1* and NIP. The *nrgg2‐1* and NIP lines had the same panicle number per plant, grain number per panicle, and seed setting ratio. No significant difference was observed in the grain and biomass yield per plant.

In contrast, the *zrgg2‐1* and *zrgg2‐2* mutants were clearly taller than the wild‐type plants (Figure 7d,e; Table 1). In addition, the 1000‐grain weights of *zrgg2‐1* and *zrgg2‐2* were 18.0% and 10.9% higher, respectively, than those of ZS97 (Figure 7f; Table 1). Other components of grain yield, including the panicle number per plant, grain number per panicle, and seed setting ratio, did not differ (Table 1). We next assessed the effects of *RGG2* on yield production. The grain yields per plant of *zrgg2‐1* and *zrgg2‐2* were 11.8% and 16.0% higher, respectively, than those of ZS97 (Figure 7g; Table 1). Because the *zrgg2‐1* and *zrgg2‐2* plants were taller than the ZS97 plants, we investigated whether *RGG2* affects plant biomass. As shown in Table 1, the biomasses of both mutants were significantly greater than those of the ZS97 plants. Taken together, these results suggest that the mutations of *RGG2* led to positive effects on grain size and yield, at least in the ZS97 background, which might potentially be useful for rice yield improvements.

**Table 1:** Major agronomic traits of ZS97 and the two *RGG2* genome‐edited variants

Traits	ZS97	*zrgg2‐1*	*zrgg2‐2*
Plant height (cm)	90.36 ± 3.82	97.72 ± 1.95**	99.62 ± 2.85**
Panicle length (cm)	22.59 ± 1.13	22.55 ± 1.01	22.98 ± 0.91
Panicle number per plant	11.54 ± 3.08	10.45 ± 2.30	11.32 ± 2.15
Grain length (mm)	7.99 ± 0.34	8.56 ± 0.35**	8.38 ± 0.34**
Grain width (mm)	3.06 ± 0.17	3.10 ± 0.16*	3.23 ± 0.16**
Grain thickness (mm)	1.86 ± 0.15	1.98 ± 0.14**	1.95 ± 0.11**
1000‐grain weight (g)	20.21 ± 0.82	23.85 ± 0.69**	22.41 ± 0.81**
Grain number per panicle	159.21 ± 27.08	162.55 ± 19.27	158.36 ± 28.08
Seed setting ratio (%)	72.33 ± 7.06	76.20 ± 5.69	74.35 ± 6.57
Grain yield per plant (g)	18.86 ± 3.48	21.09 ± 3.00*	21.87 ± 3.16**
Biomass yield per plant (g)	37.51 ± 7.35	41.66 ± 4.06*	42.94 ± 5.48**

Data are given as the means ± SD (*n* ≥ 20). Student's *t‐*test: *P <* *0.05; **P <* *0.01.

### *RGG2* negatively regulates cell expansion

**Figure 8:**
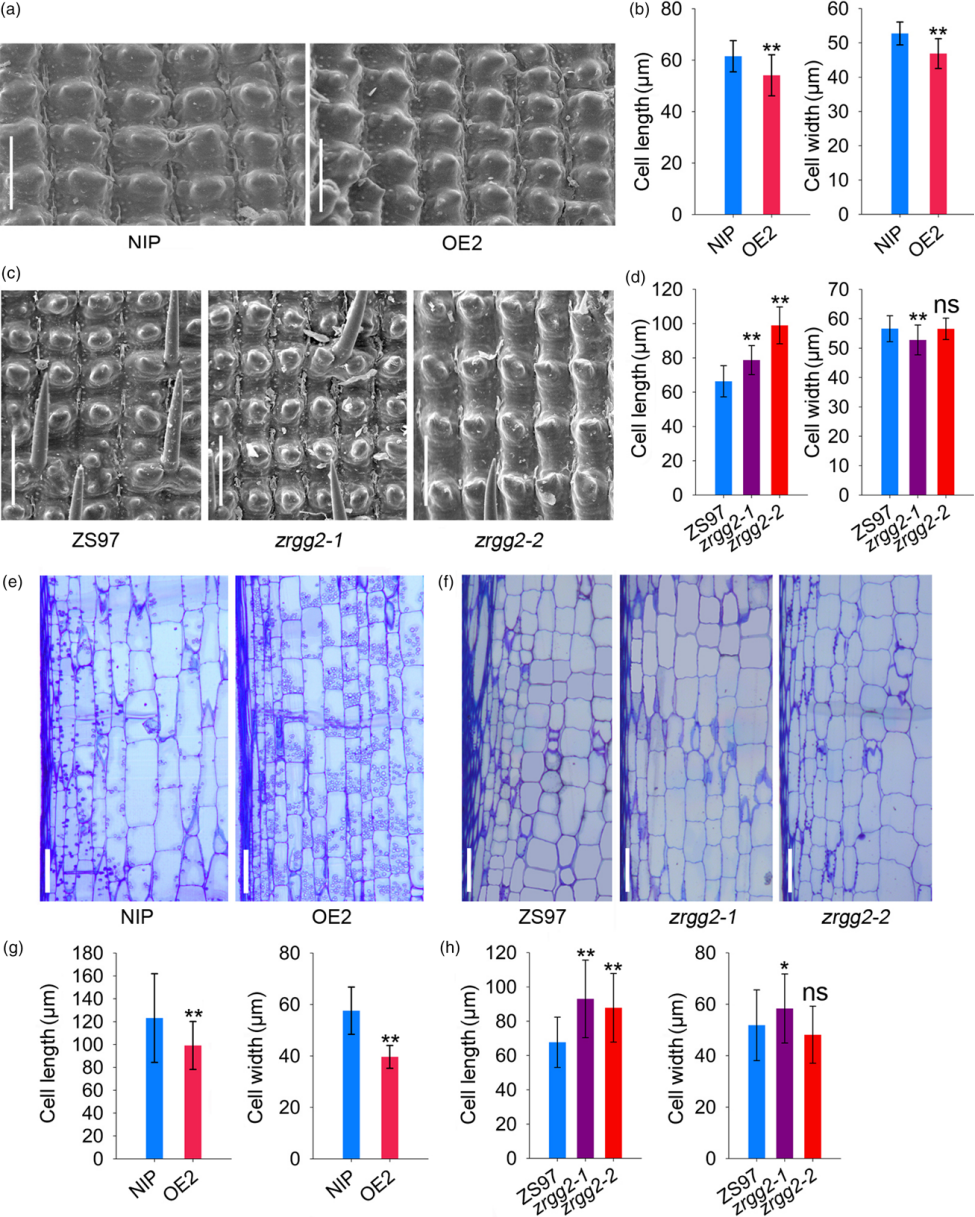
*RGG2* influences cell expansion. (a) Scanning electron microscopy analysis of the NIP and OE2 hulls. Bar = 100 μm. (b) Average length and width of the outer epidermal cells of NIP and OE2 hulls. (c) Scanning electron microscopy analysis of the hulls of ZS97, *zrgg2‐1* and *zrgg2‐2*. Bar = 100 μm. (d) Average length and width of the outer epidermal cells of ZS97, *zrgg2‐1* and *zrgg2‐2* hulls. (e) Longitudinal sections of the second internodes from the top of NIP and OE2 plants at the heading stage. Bar = 100 μm. (f) Longitudinal sections of the second internodes from the top of ZS97, *zrgg2‐1* and *zrgg2‐2* plants at the heading stage. Bar = 100 μm. (g) Cell size comparison of the second internodes from the top of NIP and OE2 plants. (h) Cell size comparison of the second internodes from the top of ZS97, *zrgg2‐1* and *zrgg2‐2* plants. Data are given as the means ± SD (*n* ≥ 20). Student's *t‐*test: *P <* *0.05; **P <* *0.01; ns, not significant.

Organ size and shape is determined by cell proliferation and cell expansion (Orozco‐Arroyo *et al*., 2015). To understand how *RGG2* influence grain size, we examined the cell number and cell size within spikelet hulls. The length and width of the outer epidermal cells of the spikelet hulls were analyzed via scanning electron microscopy. The average cell lengths of NIP and OE2 spikelet hulls were 61.5 and 54.1 μm, respectively (Figure 8a,b), and the cell width in OE2 was decreased by 11.0% (Figure 8a,b). In contrast, the cells in the spikelet hulls of *zrgg2‐1* and *zrgg2‐2* were significantly longer than those in ZS97 (Figure 8c,d). The cell width of the *zrgg2‐1* hulls was significantly shorter than that of the other two lines, and no definitive change in hull cell width was observed between *zrgg2‐2* and ZS97 (Figure 8c,d). We further compared the longitudinal sections of the second uppermost internodes at the late stage of heading. The cells of the OE2 internodes were significantly smaller than those of the NIP internodes (Figure 8e,g). The cell lengths of *zrgg2‐1* and *zrgg2‐2* were significantly increased compared to those of ZS97 (Figure 8f,h). The cells of the *zrgg2‐1* internodes were wider than those of the ZS97 internodes. However, *zrgg2‐2* and ZS97 presented the same internode cell width (Figure 8f,h). Together, these data suggest that *RGG2* regulates plant height and grain size by influencing cell expansion.

### *RGG2* is involved in the GA regulatory pathway

**Figure 9:**
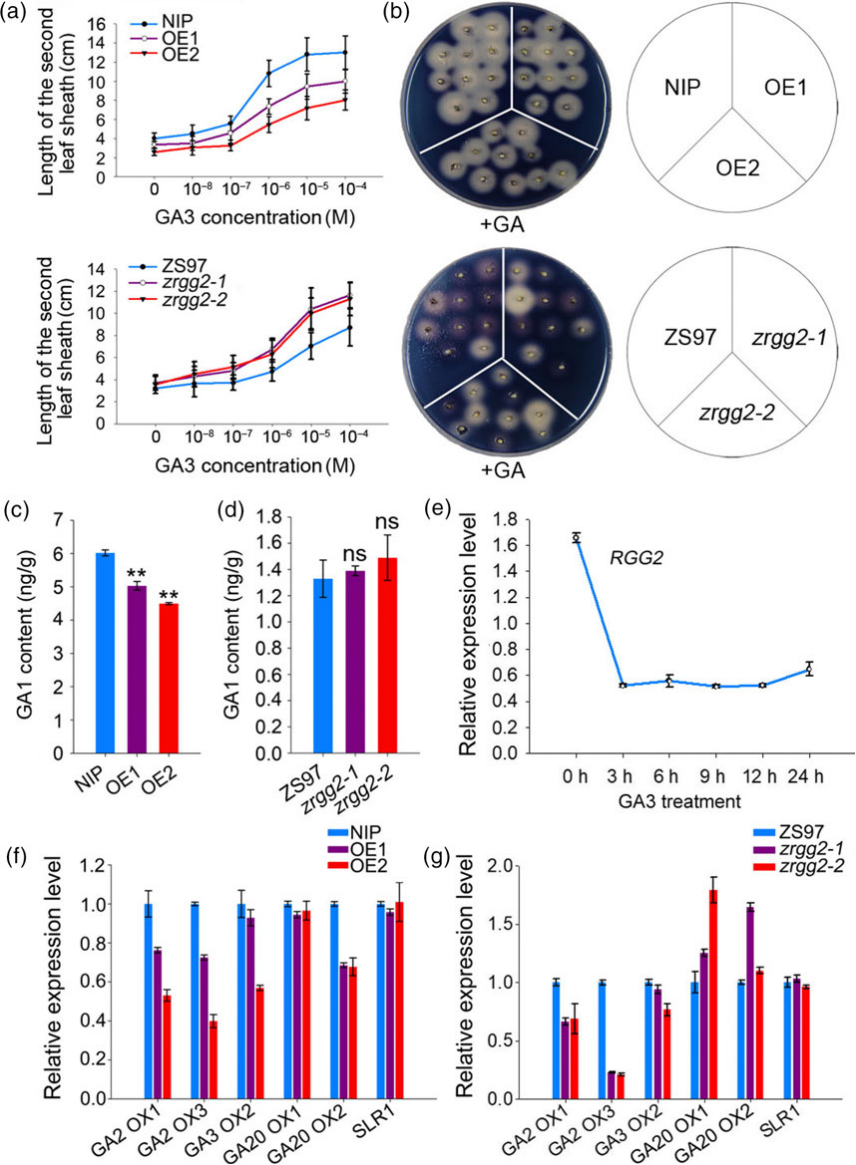
*RGG2* is involved in the GA regulation pathway. (a) Elongation of the second leaf sheath in response to GA_3_ treatment. The germinated seeds were grown in nutrient solution containing various concentrations of GA_3_ and incubated at 28 °C under 12‐h light/12‐h dark conditions. After 10 days, the lengths of the second leaf sheaths (*n* ≥ 11) were measured. (b) GA induction of α‐amylase activity in the wild‐type and transgenic lines. (c) Comparison of the GA_1_ content in the NIP, OE1 and OE2 seedlings. Student's *t‐*test: **P <* *0.01. (d) Comparison of the GA_1_ content in the ZS97, *zrgg2‐1* and *zrgg2‐2* seedlings. Student's *t‐*test: ns, not significant. (e) Expression pattern of *RGG2* under GA_3_ treatment. The NIP germinated seeds were cultivated in water. Two weeks later, the seedlings were moved to water with GA_3_ (100 μm), and the transcripts of *RGG*2 were detected via qPCR. *OsActin* was used as the reference gene. (f) qPCR analyses of the GA pathway‐related genes in the NIP, OE1 and OE2 seedlings. (g) qPCR analyses of the GA pathway‐related genes in the ZS97, *zrgg2‐1* and *zrgg2‐2* seedlings. Data are given as the means ± SD (*n* ≥ 3).

Rice dwarf mutant *d1*, which is defective in the α subunit of the heterotrimeric G protein, was originally identified as a GA signalling mutant (Ueguchi‐Tanaka *et al*., 2000). In this study, the *RGG2* overexpression plants displayed short internodes and small grains. To examine whether *RGG2* was involved in GA responses, the GA sensitivity of the transgenic lines was analyzed by measuring the second leaf sheath length after GA_3_ treatment. The leaf sheaths of OE1 and OE2 were less sensitive to GA_3_ than those of NIP (Figures 9a and S5a), whereas the leaf sheaths of *zrgg2‐1* and *zrgg2‐2* were more sensitive to GA_3_ than those of ZS97 (Figures 9a and S5b). This result was confirmed by the GA‐induced α‐amylase activity of the seeds (Figure 9b). These findings suggest that *RGG2* is involved in the response to GA in rice.

GA_1_ is the major bioactive GA that promotes the longitudinal elongation of vegetative organs in rice (Kobayashi *et al*., 1988). We measured the content of endogenous GA_1_ in the transgenic plants and found that in the OE1 and OE2 plants, the GA_1_ level decreased to approximately 83.6% and 74.6% of the NIP level, respectively (Figure 9c). However, the endogenous GA_1_ content in the *zrgg2‐1* and *zrgg2‐2* plants was not significantly different from that in the wild‐type plants (Figure 9d).

The effect of GA on *RGG2* expression was also investigated via qPCR. The expression level of *RGG2* was down‐regulated and reached the lowest level at 3 hours after the GA_3_ treatment (Figure 9e). Additionally, we used qPCR to examine the expression of GA signalling‐ and biosynthesis‐related genes. As indicated in Figure 9f, the expression levels of several GA biosynthesis pathway genes (including *OsGA20ox2*,* OsGA3ox2*,* OsGA2ox1*, and *OsGA2ox3*) in OE1 and OE2 apparently decreased compared to those in NIP. Significant changes were not observed in the expression level of *SLR1*, which is involved in GA signalling. In contrast, the transcripts of *OsGA20ox1* and *OsGA20ox2* were clearly higher in the *zrgg2‐1* and *zrgg2‐2* plants than in the ZS97 plants (Figure 9g). Together, this information suggests that *RGG2* mediates internal GA biosynthesis and is also involved in the GA signalling pathway.

### Overexpression of *RGG2* in Wuyunjing 7 (WYJ7) results in a similar phenotype to that in NIP

**Figure 10:**
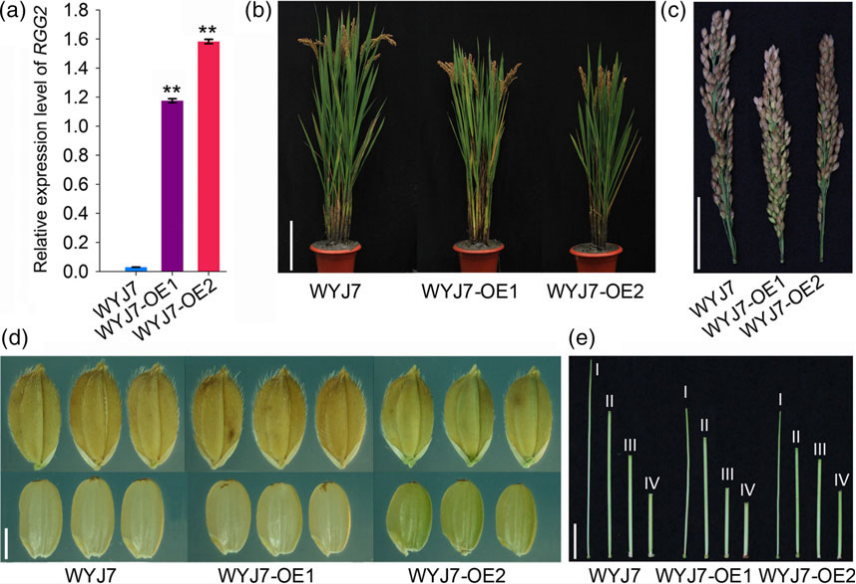
Phenotypes of the *RGG2*‐overexpressing lines in the WYJ7 background. WYJ7 is a *japonica* variety with the *qpe9‐1* allele. WYJ7‐OE1 and WYJ7‐OE2 are two overexpression lines of *RGG2* in the WYJ7 background. (a) Expression levels of *RGG2* in the WYJ7‐OE1, WYJ7‐OE2, and WYJ7 plants. (b) Plant morphology of the WYJ7 and transgenic plants. Bar = 20 cm. (c) Panicle architecture of the WYJ7 and transgenic plants. Bar = 5 cm. (d) Grains and brown rice of WYJ7 and the transgenic plants. Bar = 2 mm. (e) The first internode (I: panicle‐neck internode) to fourth internode (IV: basal internode) of the WYJ7 and transgenic plants. Bar = 5 cm.

We previously showed that NIP is a *japonica* variety that carries a functional *qPE9‐1* allele (Zhou *et al*., 2009). To investigate whether *RGG2* functions in a *qPE9‐1*/*DEP1* mutant background, we introduced the *RGG2*‐overexpressing construct into WYJ7, a high‐yield *japonica* variety that harbours the *qPE9‐1/DEP1* allele. The *RGG2*‐overexpressing WYJ7 plants exhibited dwarfism and had both shorter panicles and smaller grains, and the phenotypes were similar to those of the NIP transgenic plants (Figure 10; Table S3). These results suggest that overexpression of *RGG2* has similar effects on plant height and grain size regardless of the allelic status of *qPE9‐1/DEP1* gene and imply that the genetic effects of different G_γ_‐encoding genes could be pyramided to regulate grain size.

## Discussion

To date, the biological roles of G_γ_‐encoding genes have not been fully characterized in plants. The function of G_γ_ was initially restricted to anchoring G_βγ_ dimers to the membrane. Now, G_γ_ subunits are considered as important components of heterotrimers that regulate multiple roles in growth and development by forming independent signal‐transducing G_βγ_ dimers. G_γ_ subunits can be divided into three clades: type A, type B and type C. Previously, only two type A G_γ_ subunit genes, *AGG1* and *AGG2*, were thought to exist in Arabidopsis. However, AGG3, which modulates both morphological development and the ABA regulation of stomatal aperture, was identified as a new Arabidopsis G protein γ subunit (Chakravorty *et al*., 2011). AGG3, a type C G_γ_, represents a novel class that is widespread throughout the plant kingdom but non‐existent in animals (Chakravorty *et al*., 2011; Trusov *et al*., 2012). In rice, the *AGG3* homologues *GS3* and *qPE9‐1/DEP1* were originally identified as important QTLs for grain size and yield, although their identity as G_γ_ subunits was unknown at that time because of the atypical nature of these proteins (Chakravorty *et al*., 2011; Fan *et al*., 2006; Huang *et al*., 2009; Zhou *et al*., 2009). Although RGG2 has long been identified as a component of the rice G protein complex (Kato *et al*., 2004), its function remains unknown.

Phylogenetic analysis revealed that rice RGG1 is a type A G_γ_ protein while RGG2 is a member of the type B class (Figure 1). Both *RGG1* and *RGG2* were constitutively expressed (Figure 3). However, the expression levels of *RGG2* were significantly higher than those of *RGG1*,* GS3*,* qPE9‐1*/*DEP1* and *GGC2* in all tissues examined, and this finding was similar to the expression patterns of G_γ_ genes in tomato (Subramaniam *et al*., 2016). These results suggest that *RGG2* may play an important role in rice development. In this study, we focused on the molecular characterization and biological function of the only type B subunit in the model crop rice. Transient expression in rice protoplasts and tobacco leaves clearly indicated that the RGG2 protein is localized to the nucleus, cytoplasm and plasma membrane (Figure 4), although the RGB1, RGG1 and RGG2 proteins were previously reported to localize to the plasma membrane (Kato *et al*., 2004). The potential nuclear localization or translocation of the G_βγ_ dimer in mammalian cells was recently reported, and this dimer colocalizes with the AP‐1 transcription factor and recruits histone deacetylases to inhibit AP‐1 transcriptional activity (Chang *et al*., 2013; Robitaille *et al*., 2010). More recently, Liu *et al*. (2018) detected RGB1‐GFP and GS3‐GFP fusion proteins in both the plasma membrane and nucleus in rice and found that the nuclear translocation of DEP1/qPE9‐1 is involved in G protein signalling. Conventional type A G_γ_ subunits are membrane localized because of the C‐terminal CaaX motifs, which are essential for plasma membrane targeting (Zeng *et al*., 2007), and all 12 human G_γ_ subunits are membrane associated. Given the lack of a CaaX motif and transmembrane domain, the localization of RGG2 to the nucleus and cytoplasm is not surprising. A set of truncated AGG3 proteins was recently generated for subcellular localization analysis (Wolfenstetter *et al*., 2015). Neither the absence of the transmembrane domain nor the mutation of the C‐terminal CaaX motif affected the plasma membrane localization of AGG3. Only the deletion of both the transmembrane domain and the C‐terminus caused redistribution of the AGG3 mutant protein to the cytoplasm (Wolfenstetter *et al*., 2015). Structurally, the RGG2 subunit is similar to the truncated AGG3 protein, because RGG2 lacks both the transmembrane domain and the C‐terminus. The type B G_γ_ subunit in tomato, SlGGB1, also localizes to the nucleus, plasma membrane and cytoplasm (Subramaniam *et al*., 2016), thus sharing a common localization pattern with RGG2. These results indicate that both the transmembrane domain and CaaX motif might be sufficient but not essential for plasma membrane localization of G_γ_ protein in plants.

To investigate the function of the type B G_γ_ protein in rice, we successfully generated transgenic plants overexpressing *RGG2*. The OE1 and OE2 plants presented a semidwarf and compact plant architecture as well as short panicles and small grains (Figures 5 and 6), which is consistent with previous reports (Sun *et al*., 2014). Other important elements, including the number of panicles, grain number per panicle and seed setting ratio, were not changed. As a result, the grain yield and biomass yield per plant of OE1 and OE2 were decreased (Figure 5). CRISPR/Cas9 is a novel tool for targeted mutagenesis and applicable to rice. By applying CRISPR/Cas9‐mediated targeted mutagenesis, we successfully generated one homozygous mutant in the NIP background (*nrgg2‐1*) and two homozygous mutants in the ZS97 background (*zrgg2‐1* and *zrgg2‐2*). The mutation of *RGG2* in the NIP background caused slightly increased grain length and weight but had no effect on grain yield (Table S2). The *nrgg2‐1* mutant had a 6‐bp deletion at its target site that caused a three‐amino acid mutation (Figure S3). Those three amino acids are not conserved in the GGL domain (Figure S1), which could possibly explain the weak phenotypic variation in the *nrgg2‐1* mutant. Interestingly, we did not obtain knockout mutant of *RGG2* in both NIP and ZS97 backgrounds although lots of screening were performed. We also compared the expression levels of several reported genes for rice inflorescence and grain development, such as *SNB* (Lee and An, 2012), *PAP2* (Kobayashi *et al*., 2010), *EP2* (Zhu *et al*., 2010), *LAX1* (Komatsu *et al*., 2003), *LAX2* (Tabuchi *et al*., 2011), and *IPA1* (Lu *et al*., 2013) between wild‐type and the transgenic lines (Figure S6). No significant difference was found, indicating that *RGG2* regulates inflorescence and grain development though an independent pathway.

The *zrgg2‐1* and *zrgg2‐2* plants, having more amino acid mutations than the *nrgg2‐1* mutant, produced clearly larger and heavier grains (Figure 7f; Table 1). Scanning electron microscopy revealed that the outer epidermal cells of the spikelet hulls of *zrgg2‐1* and *zrgg2‐2* were much longer than those of ZS97 (Figure 8). Despite their greatly enlarged grain size, *zrgg2‐1* and *zrgg2‐2* also exhibited enhanced growth. Both mutants were clearly taller than the ZS97 plants (Table 1), although differences in panicle number per plant, grain number per panicle and the seed setting ratio were not observed. The field test results showed that the mutation of *RGG2* increased the grain yield per plant by 11.8% and 16.0% in the ZS97 background (Table 1). These data suggest that *RGG2* plays a negative role in plant growth and yield production and that manipulation of *RGG2* can increase the plant biomass, grain weight and yield in rice. Moreover, genomic synteny widely exists in grass species (Doust *et al*., 2005; Zhang and Yuan, 2014). For instance, the *CLAVATA* (*CLV*) signalling responsible for inflorescence development was partially conserved in grasses. The investigation of homologues of *RGG2* gene in grasses will provide a new strategy for yield improvement.

According to the established concept of the heterotrimeric G protein, the G_β_ and G_γ_ subunits act as heterodimers, which means they function together. For instance, the Arabidopsis triple mutant *agg1agg2agg3* recapitulates the *agb1* phenotypes, including the reduced flower and silique sizes (Thung *et al*., 2012). We previously reported that overexpression of *qPE9‐1*/*DEP1* results in increased grain size and elongated internodes and panicles (Zhou *et al*., 2009). Plants overexpressing *GS3* produce relatively short grains, whereas the *GS3*‐loss‐of‐function plants produce relatively long grains and increased yield (Fan *et al*., 2006; Mao *et al*., 2010). In this study, *RGG2* acts as a negative regulator of grain size and yield production. These results imply that the molecular mechanism of the G_γ_ subunits in rice may differ from that in Arabidopsis. *qPE9‐1*/*DEP1* displays functional divergence with *GS3* and *RGG2* in rice. *RGG2* appears to have similar genetic effects as *GS3* on agriculturally relevant rice plant phenotypes. Interestingly, both the transcripts of *GS3* and *RGG2* decreased as the inflorescence and spikelet elongation (Figure S2). Very recently, Sun *et al*. (2018) characterized the function of GGC2, a controversial G_γ_ subunit in rice, and proposed a genetic model depicting the pathway of the G proteins involved in rice grain size regulation. In this model, DEP1, GGC2 and GS3 antagonistically regulate grain size. DEP1 and GGC2, individually or in combination, increase grain length when complexed with G_β_. GS3, which has no effect on grain size by itself, reduces grain length by competitively interacting with G_β_. According to this model, RGG2 may negatively regulate grain size via the occupation of RGB1 and the consequent disruption of the RGB1‐DEP1 and RGB1‐GGC2 dimers.

**Figure 11:**
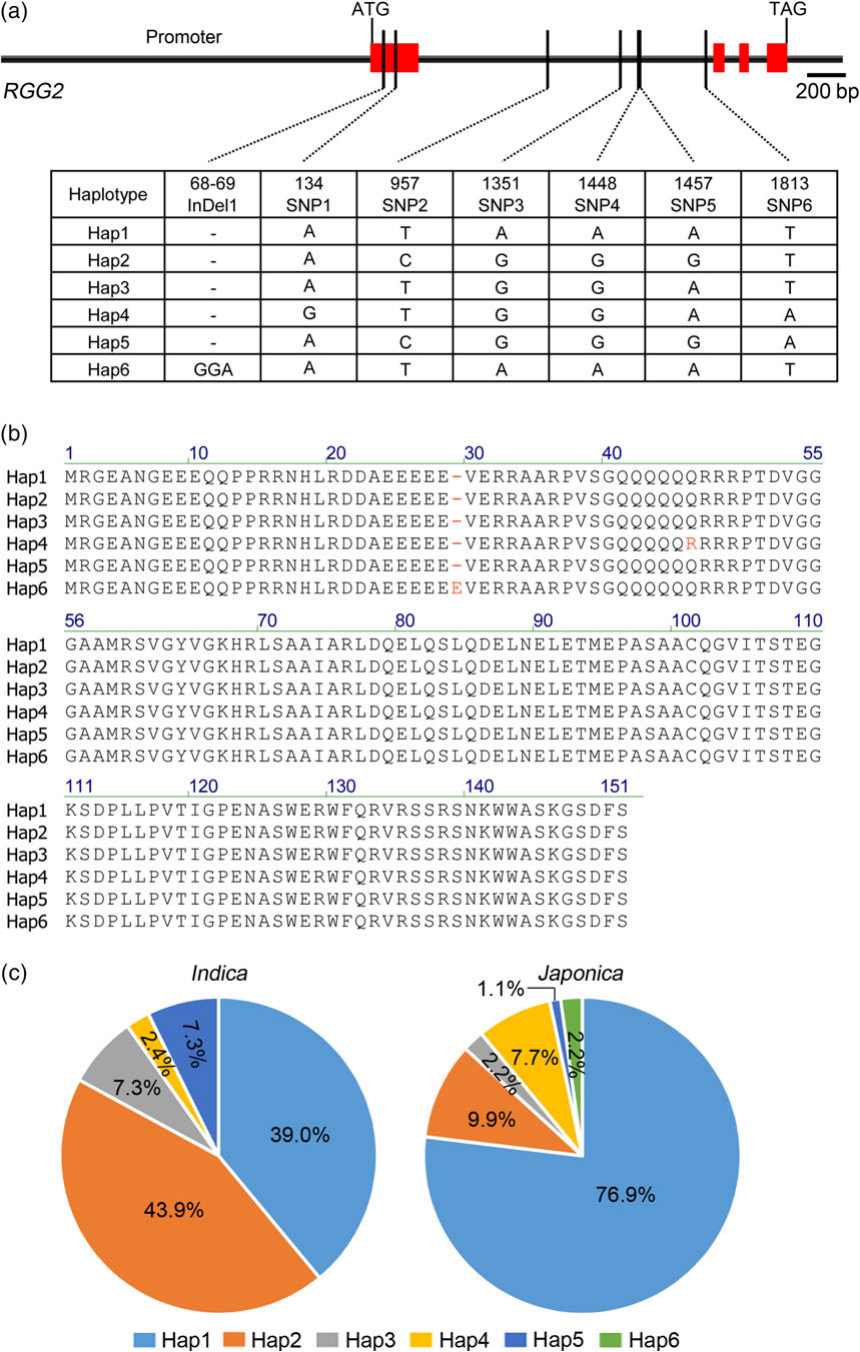
Different haplotypes of *RGG2* in 132 rice germplasms. (a) Six haplotypes distributed in the 4.4‐kb genomic DNA of *RGG2*. Red represents the exons of *RGG2*. Bar = 200 bp. (b) Amino acid comparison of the six haplotypes. SNP1 caused one amino acid change (from Gln to Arg). Indel1 caused one amino acid Glu insert. (c) Percentages of six haplotypes in 41 *indica* and 91 *japonica* germplasms.

To identify possible natural variations of *RGG2*, we sequenced and analyzed the genomic DNA fragment of *RGG2* (including ~1.9‐Kb promoter region upstream of the start codon) in 132 rice germplasms (Table S5). The haplotype analysis based on the genomic sequence yielded a total of 6 SNPs and one indel representing six haplotypes (Figure 11a). Polymorphisms were not observed in the promoter region. Only Indel1 and SNP1 were located in the exon, and they led to amino acid changes, with Indel1 causing the insertion of one amino acid (Glu) and SNP1 causing the change of one amino acid (from Gln to Arg) (Figure 11b). According to the sequencing results, Hap1 and Hap2 were mostly distributed in *japonica* and *indica* rice, respectively (Figure 11c). NIP carries Hap1, while ZS97 carries Hap2 (Table S5). However, they have the same RGG2 protein sequence. One rare haplotype containing Indel1 (Hap6) was found in Suyunuo and Dalijing. These two germplasms produce extremely large grains (Table S5), which imply that Hap6 has beneficial effects on grain size and weight. However, the genetic effect and breeding value of the rare *RGG2* allele needs to be further investigated.

## Materials and methods

### Plant materials

Three wild‐type varieties, NIP, WYJ7 and ZS97, were used in this study. NIP and WYJ7 are two *japonica* rice varieties while ZS97 is an *indica* rice variety. To generate transgenic rice plants overexpressing *RGG2* (LOC_Os02g04520), the coding region was amplified from first‐strand cDNA. The PCR product was then inserted into a p1301UN vector along with the maize *UBIQUITIN* (*Ubi*) promoter and nopaline synthase (NOS) terminator. The overexpression construct was then transferred into both NIP and WYJ7. Individual transgenic plants were selected by their resistance to 75 mg/l hygromycin in half‐strength Murashige and Skoog medium. The resistant plants were subsequently transplanted to the field. Homozygous T_2_‐ and T_3_‐generation plants and their seeds were used for further experiments. The *RGG2* promoter, an approximately 1.8‐kb DNA fragment upstream of the translation start site, was amplified and subcloned into a pCAMBIA1301 vector to generate a promoter‐GUS fusion construct for the expression pattern analysis. The mutants of *RGG2* were generated using the CRISPR/Cas9 system (Wang *et al*., 2015). A target sequence located within the GGL domain of *RGG2* was selected and cloned into an SK‐gRNA vector. The single gRNA including the target gene was then inserted into pC1300‐Cas9, which is a CRISPR/Cas9 binary vector driven by two CaMV 35S promoters of expression in rice. The resultant CRISPR/Cas9 knockout plasmid was then transferred into NIP and ZS97. Among the plants of the T_0_ generation, the various types of *RGG2* were confirmed by sequencing (Table S4). Homozygous lines were subsequently grown for agronomic trait analysis.

All constructs were transformed into *A. tumefaciens* strain EHA105. For transformation, calli were induced from mature seeds and then transformed via *A. tumefaciens*‐mediated transformation (Hiei *et al*., 1994).

### Plant growth conditions and measurements of agronomic traits

The wild‐type and transgenic plants were grown in an experimental field of Yangzhou University (E119°25′/N32°23′) from May through October in 2015, 2016, 2017 and 2018. Each line was grown as three replicates in paddy fields, with each plot containing 40~60 plants. The distance between the plants within a row was 16.7 cm, and the distance between the rows was 23.3 cm. Field management and disease and pest control were performed in accordance with standard procedures to prevent yield loss during the growth period. The plant height and panicle number per plant were determined at the maturation period. The grain length, width and thickness as well as the grain number per panicle were determined when the seeds were harvested. The biomass and seed weight were measured after harvest, and the dry mature plants and seeds were maintained at 37 °C for 1 week before weighing.

### GA induction in second leaf sheath elongation and α‐amylase activity

The germinated seeds were grown in the solution that contained various concentrations of GA_3_ (Solarbio, China) and incubated at 28 °C under 12‐h light/12‐h dark conditions. After 10 days, the length of the second leaf sheaths was measured. To assay the α‐amylase activity, embryoless half‐seeds of the wild‐type and transgenic lines were sterilized with 2% NaClO, and then they were washed five times with sterilized water. The embryoless half‐seeds were placed on plates with 2% potato dextrose agar (Solarbio, China) containing 1 μm GA_3_, and then the plates were incubated at 25 °C in darkness. After 60 h, the plates were subjected to I_2_ gas and subsequently photographed.

### Yeast two‐hybrid assays of protein interactions

To assay the interactions between different RGG2 domains and the RGB1 protein, the RGG2 protein as well as truncated proteins were cloned into a pGBKT_7_‐BD vector and the RGB1 protein was inserted into a pGADT_7_‐AD vector (Clontech, Mountain View, CA). Yeast two‐hybrid assays were then performed in accordance with the manufacturer's instructions.

### Co‐immunoprecipitation and LC‐MS/MS assays

Full‐length cDNA of the *RGB1* gene was amplified from NIP and cloned into a modified pEGAD vector with the maize ubiquitin promoter. The UBI::GFP‐RGB1 and UBI::GFP constructs were then transformed into NIP, and the obtained transgenic rice was used for co‐immunoprecipitation assay. To verify the interaction of RGG2 and RGB1, total proteins were extracted from seedlings of UBI::GFP and UBI::GFP‐RGB1 transgenic rice via treatment with 50 mm Tris (pH 7.5), 0.1% IGEPAL CA‐630, 150 mm NaCl, Phosphatase Inhibitor Cocktail and Proteinase Inhibitor Cocktail (Roche, Switzerland). The soluble proteins were incubated with anti‐GFP magnetic beads (MBL, Japan) at 4 °C for at least 12 h. The magnetic beads were washed 4~5 times with PBS buffer and then eluted with 1 m glycine. The immunoprecipitates were separated via SDS‐PAGE, and the RGG2 protein was detected via western blotting with anti‐RGG2 antibody. To further verify the RGG2 interaction with RGB1, the immunoprecipitated proteins eluted from anti‐GFP magnetic beads were subjected to Liquid Chromatography‐Mass Spectrometry/Mass Spectrometry (LC‐MS/MS) assays by Wuhan GeneCreate Biological Engineering Co., Ltd, China.

### RNA preparation and qPCR analysis

Total rice RNA was extracted with an RNA Prep Pure Kit (Tiangen, Beijing, China) in accordance with the manufacturer's instructions and then treated with DNase to remove any genomic DNA. Complementary DNA was synthesized from 1 μg of total RNA using a reverse transcription kit (Tiangen, Beijing, China). The primer pairs used for the qPCR are listed in Table S4. The primers of the GA pathway‐related genes were the same as those described previously (Tong *et al*., 2014; Wang *et al*., 2009). To confirm that the expression of *RGG2* was regulated by GA, 14‐d‐old hydroponically cultured NIP seedlings placed into 100 μm GA_3_ (Solarbio, Beijing, China) were used. The expression levels of *RGG2* at 0, 3, 6, 9, 12 and 24 h after treatment with GA_3_ were detected via qPCR. The rice *OsActin* gene (LOC_Os03g50885) was used as an internal control. The qPCR analysis was performed in a total volume of 25 μL, which consisted of 2 μL of cDNA, 0.2 mm of each primer, and 12.5 μL of 2X SYBR Green PCR Master Mix (Takara, Japan). The qPCR assay was conducted using a real‐time quantitative PCR system (ViiA7, Applied Biosystems, Foster City, CA) using the following programme: 95 °C for 3 min followed by 40 cycles of 94 °C for 30 s, 55 °C for 30 s and 72 °C for 40 s. The data were presented as the mean ± SD of three replicates. The relative gene expression was calculated using the 2^−ΔΔCT^ method.

### GUS histochemical staining

GUS histochemical staining was performed at 37 °C as described by Jefferson (1987). The reaction was stopped by adding ethanol, after which the samples were treated with fresh 70% ethanol several times until the plant tissues were mostly discolored. Subsequently, the samples were observed and photographed under a microscope (DM1000, Leica, Germany).

### Subcellular localization

For the subcellular localization analysis, the coding region of *RGG2* was fused to GFP in frame in a *p163‐GFP* vector to generate *CaMV35S::RGG2‐GFP*; and *CaMV35S::GFP* was used as a control. These two constructs were transferred into rice NIP protoplasts. The transformed protoplasts were observed and photographed using a confocal microscope (LSM 710, Zeiss, Germany) 1 day after transformation. For tobacco transient expression, the full‐length cDNA of *RGG2* from NIP was amplified and inserted into a pEGAD vector. The pEGAD‐RGG2 and P19 vectors were cotransformed into *A. tumefaciens* strain GV3101, after which the bacteria were incubated at 28 °C under shaking to OD_600_ = 0.7–1.0. Transformation solution (1 mL of 1 m morpholineethanesulfonic acid, 1 mL of 1 m MgCl_2_, 100 μL of 0.2 m acetosyringone, and 98 mL of ddH_2_O) containing pEGAD‐RGG2 was injected into 5‐week‐old tobacco leaves, and fluorescence was observed 3 days after injection.

### Morphological and cellular analyses

Fresh spikelets from the panicles at heading stage were observed directly with a scanning electron microscope (XL‐30ESEM, Philips, Holland). With respect to histology, the spikelet hulls and second internodes were fixed in 2% glutaraldehyde, dehydrated in a graded ethanol series, and embedded in Spurr resin. The longitudinal sections of the internodes were produced using an ultramicrotome (EM UC7, Leica, Germany), and then the sections were stained with 0.5% toluidine blue and observed using a microscope (DM1000, Leica, Germany). The cell size of the spikelet hulls and internodes was measured using ImageJ software (National Institutes of Health, Maryland, USA).

### Phylogenetic analysis

The sequences of the Arabidopsis, rice and tomato G_γ_ proteins were acquired from the Phytozome database (https://phytozome.jgi.doe.gov/pz/portal.html) and the Rice Functional Genomics and Breeding Database (RFDB; http://www.rmbreeding.cn/index.php). Multiple alignments of these selected sequences were performed with ClustalX. The sequence identities between proteins were calculated and visualized using GeneDoc software (http://genedoc.software.informer.com/2.7/). Phylogenetic analysis was performed using both the maximum likelihood (ML) and neighbor‐joining (NJ) methods with MEGA version 7.0. The Jones‐Taylor‐Thornton (JTT) model was used to construct both ML and NJ trees. A total of 100 nonparametric bootstrap samplings were performed to estimate the support level for each internal branch for both the ML and NJ trees.

### Statistical analysis

All numerical data are presented as the means ± SD (the error bars indicate the standard deviation of the mean). Statistical analysis was conducted by comparing the raw data of all individuals of each transgenic line with those of the wild‐type line using SigmaPlot software (Systat Software Inc., California, USA). Differences between the transgenic lines and wild‐type lines were then compared. Significance levels were determined according to Student's *t*‐test: *P <* *0.05, **P <* *0.01, ns not significant.

### Primers

The nucleotides of all primers used for vector construction as well as the PCR and qPCR analyses are provided in Table S4.

## Conflict of interest

The authors declare no conflicts of interest.

## Supporting information


**Figure S1** Alignment of the G_γ_ proteins in rice, Arabidopsis and tomato.
**Figure S2** Expression analysis of *RGG2* (a) and *GS3* (b) in the NIP panicles at 2‐cm (YP2), 7‐cm (YP7), 14‐cm (YP14), 18‐cm (YP18), 20‐cm (YP20), 21‐cm (YP21), and 24‐cm (YP24) stages.
**Figure S3** Targeted mutagenesis of the *RGG2* gene under the NIP background using a CRISPR/Cas9 system.
**Figure S4** Comparison of plant and grain phenotypes between NIP and the *nrgg2‐1* mutant.
**Figure S5** Seedling growth phenotypes of the wild‐type and transgenic lines under different GA_3_ concentrations.
**Figure S6** Expression levels of several reported genes for rice inflorescence and grain development between the wild‐type and transgenic plants.
**Table S1** Identity between the sequences of the Arabidopsis, rice and tomato G_γ_ proteins.
**Table S2** Major agronomic traits of NIP and *nrgg2‐1*.
**Table S3** Comparison of major agronomic traits between WYJ7 and the two overexpression lines of *RGG2*.
**Table S4** Primers used in this study.
**Table S5** Different haplotypes of *RGG2* in 132 rice germplasms.
