# Supplementary material for: Mutation of RGG2, which encodes a type B heterotrimeric G protein γ subunit, increases grain size and yield production in rice
Source: Plant Biotechnol J. 2018 Dec 13;17(3):650–64. doi: 10.1111/pbi.13005 (PMC6381795; doi:10.1111/pbi.13005)
Supplement: Supplementary file 1 — Figure S1 Alignment of the Gγ proteins in rice, Arabidopsis and tomato. Figure S2 Expression analysis of RGG2 (a) and GS3 (b) in the NIP panicles at 2‐cm (YP2), 7‐cm (YP7), 14‐cm (YP14), 18‐cm (YP18), 20‐cm (YP20), 21‐cm (YP21), and 24‐cm (YP24) stages. Figure S3 Targeted mutagenesis of the RGG2 gene under the NIP background using a CRISPR/Cas9 system. Figure S4 Comparison of plant and grain phenotypes between NIP and the nrgg2‐1 mutant. Figure S5 Seedling growth phenotypes of the wild‐type and transgenic lines under different GA3 concentrations. Figure S6 Expression levels of several reported genes for rice inflorescence and grain development between the wild‐type and transgenic plants. Table S1 Identity between the sequences of the Arabidopsis, rice and tomato Gγ proteins. Table S2 Major agronomic traits of NIP and nrgg2‐1. Table S3 Comparison of major agronomic traits between WYJ7 and the two overexpression lines of RGG2. Table S4 Primers used in this study. Table S5 Different haplotypes of RGG2 in 132 rice germplasms. [file PBI-17-650-s001.docx]

**Figure S1** Alignment of the G_γ_ proteins in rice, Arabidopsis and tomato. The putative GGL domain is indicated by a black line. The CaaX and SDFS motifs are indicated in black and red boxes, respectively.

**
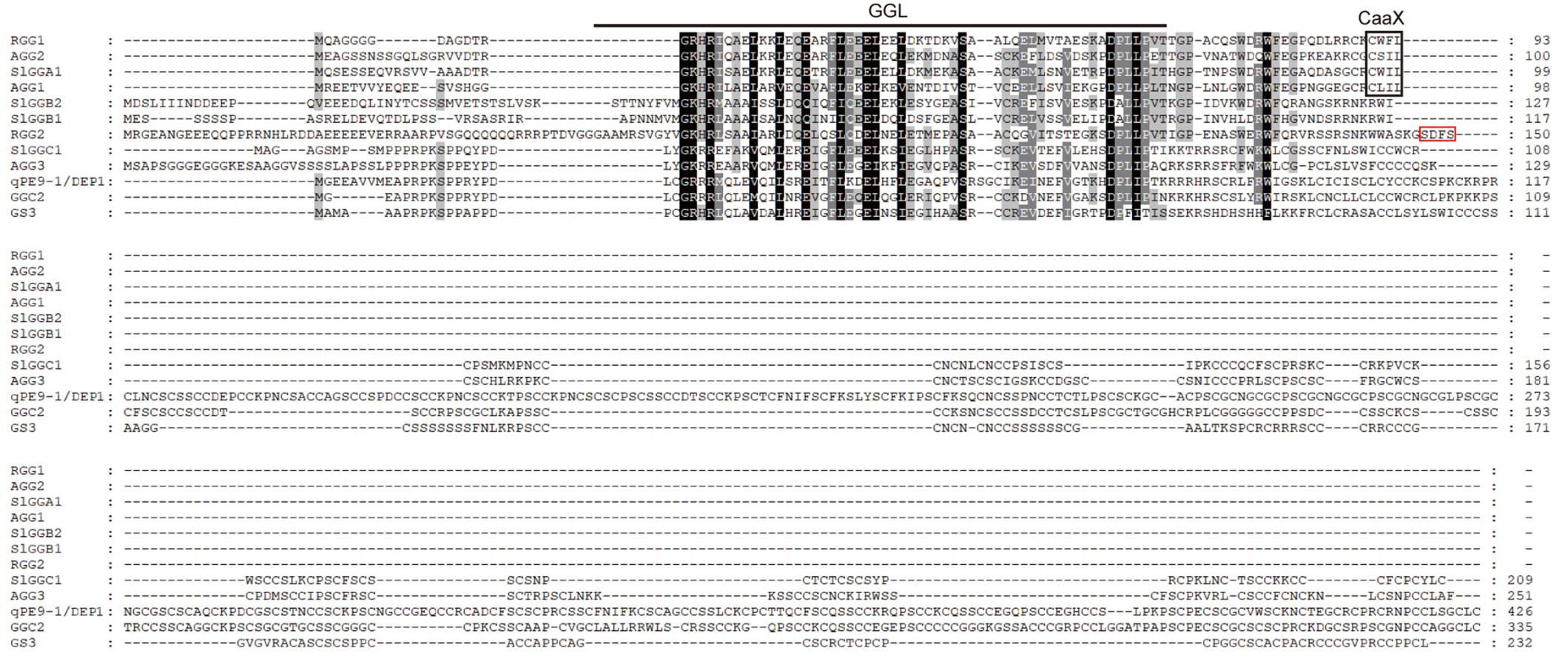
**

**Figure S2** Quantitative expression analysis of *RGG2* (a) and *GS3* (b) in the NIP panicles at 2-cm (YP2), 7-cm (YP7), 14-cm (YP14), 18-cm (YP18), 20-cm (YP20), 21-cm (YP21), and 24-cm (YP24) stages. The expression level of the rice *OsActin* gene was amplified as a control. Values are means ± S.E. of three independent experiments.

**
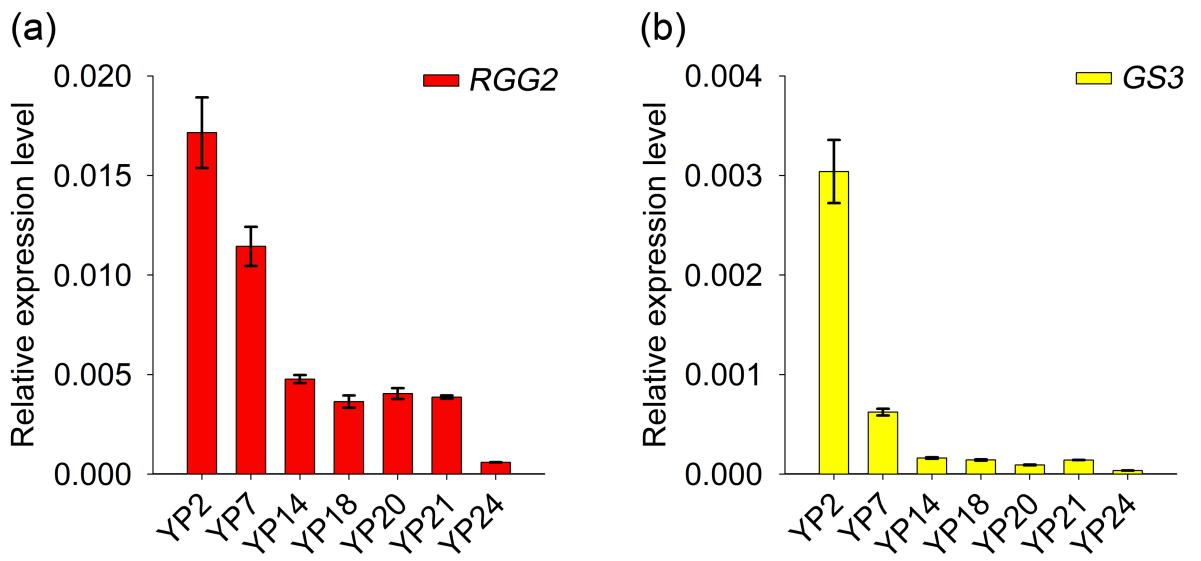
**

**Figure S3** Targeted mutagenesis of the *RGG2* gene under the NIP background using the CRISPR/Cas9 system. A mutation event was generated by CRISPR/Cas9 in NIP and confirmed by sequencing. Three amino acid changes in the RGG2 protein of the *nrgg2-1* mutant. The red rectangles indicate the GGL domain.


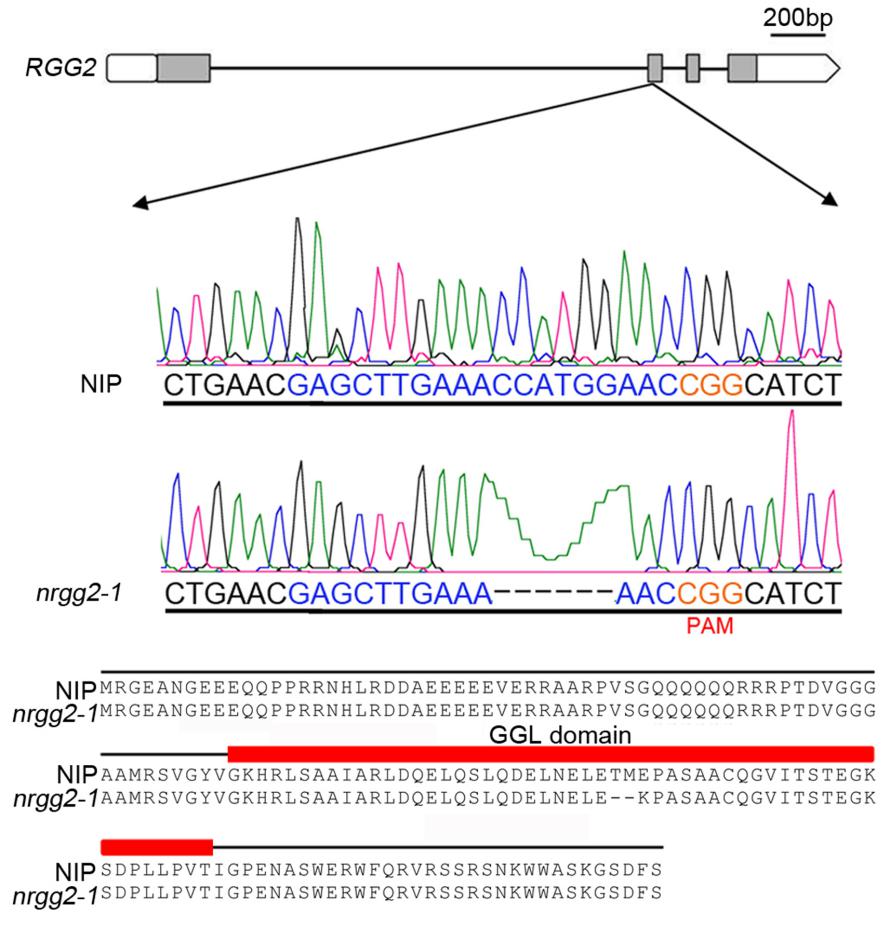


**Figure S4** Comparison of plant and grain phenotypes between NIP and the *nrgg2-1* mutant. (a) Plant architecture of NIP and *nrgg2-1*. Bar = 20 cm. (b) Panicles of NIP and *nrgg2-1*. Bar = 5 cm. (c) Grains and brown rice of NIP and *nrgg2-1*. Bars = 2 mm.


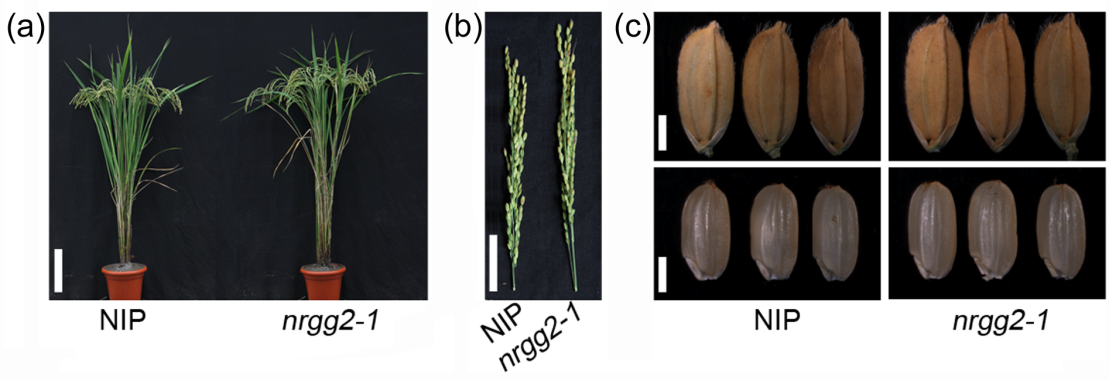


**Figure S5** Seedling growth phenotype of wild-type and transgenic lines under different GA_3_ treatment concentrations. (a) Seedlings of NIP, OE1 and OE2. (b) Seedlings of ZS97, *zrgg2-1* and *zrgg2-2*. The germinated seeds were grown in nutrient solution containing various concentrations of GA_3_ and incubated at 28°C under 12-h light/12-h dark conditions. After 10 days, the seedlings were photographed. Bars = 5 cm.


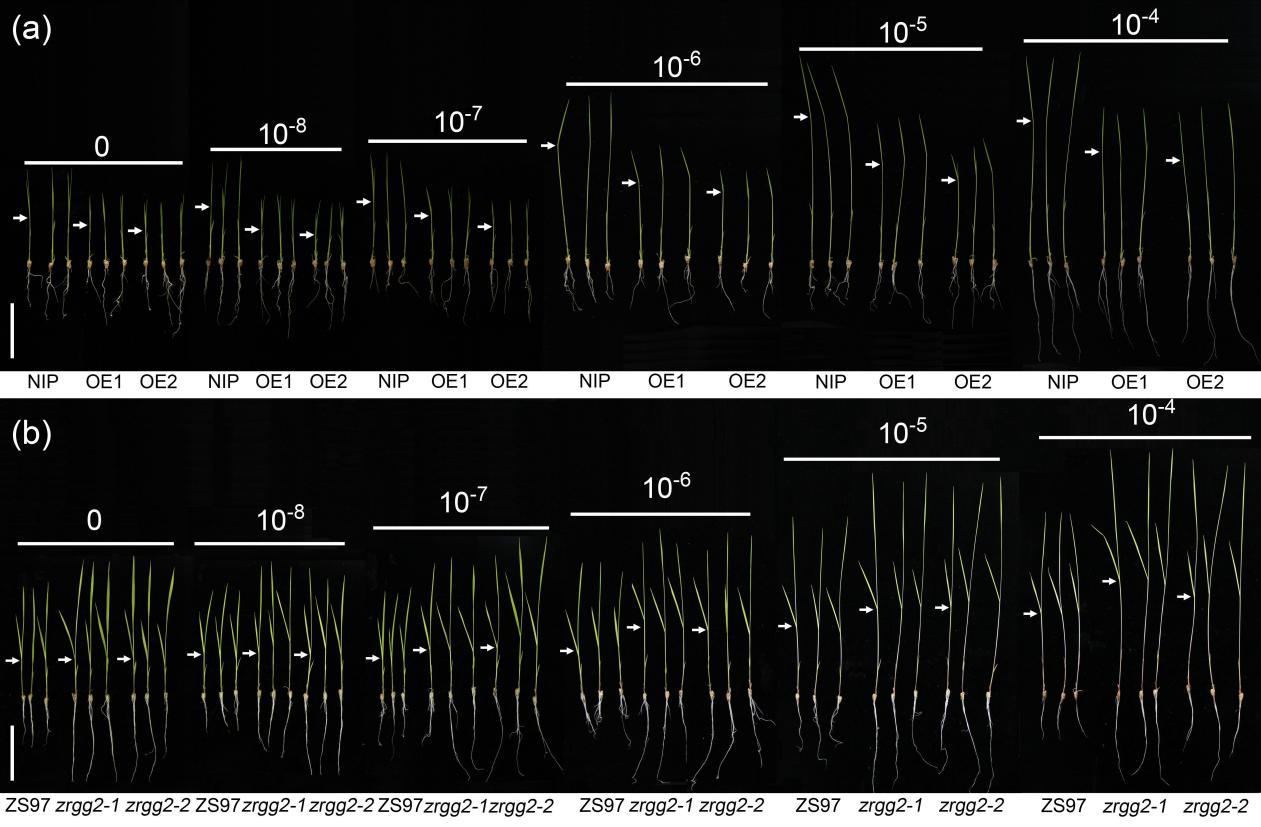


**Figure S6** Expression levels of several reported genes for rice inflorescence and grain development between wild-type and transgenic plants. Young panicles were selected for expression analysis. The relative expression levels of each gene were normalized by *OsActin*. Values are means ± S.E. of three independent experiments.


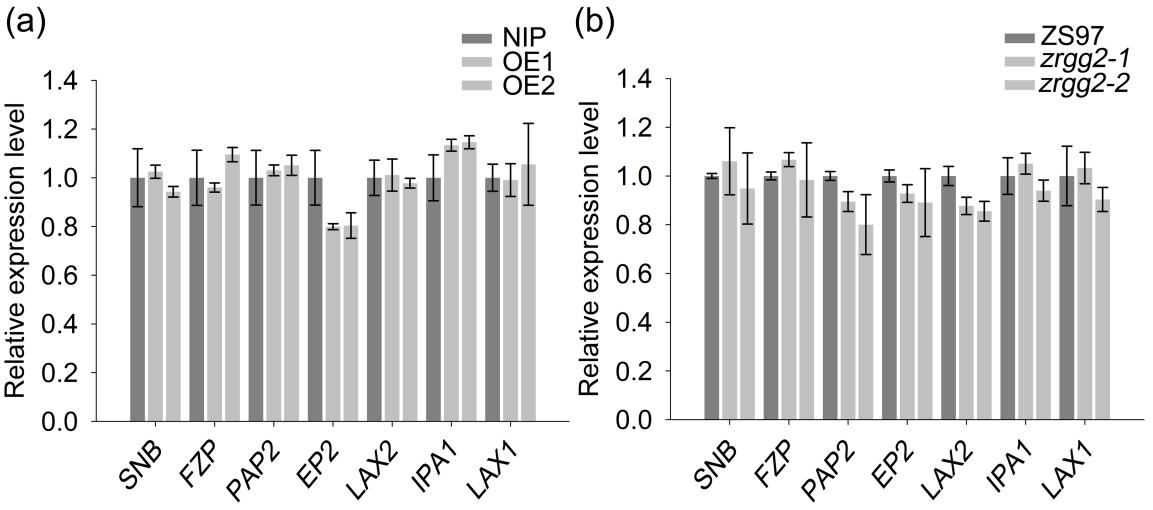


**Table S1** Identity between the sequences of the Arabidopsis, rice and tomato G_γ_ proteins.

|  | AGG2 | SlGGA1 | AGG1 | SlGGB2 | SlGGB1 | RGG2 | SlGGC1 | AGG3 | qPE9-1/DEP1 | GGC2 | GS3 |
| --- | --- | --- | --- | --- | --- | --- | --- | --- | --- | --- | --- |
| RGG1 | 56% | 57% | 43% | 26% | 27% | 24% | 9% | 7% | 5% | 7% | 8% |
|  | 66% | 64% | 53% | 42% | 38% | 46% | 33% | 29% | 35% | 37% | 27% |
| AGG2 |  | 60% | 46% | 32% | 29% | 23% | 12% | 9% | 5% | 7% | 8% |
|  |  | 74% | 55% | 51% | 44% | 50% | 46% | 40% | 37% | 40% | 33% |
| SlGGA1 |  |  | 48% | 29% | 30% | 26% | 11% | 9% | 5% | 7% | 8% |
|  |  |  | 55% | 48% | 46% | 51% | 42% | 37% | 33% | 37% | 31% |
| AGG1 |  |  |  | 28% | 28% | 22% | 10% | 8% | 5% | 6% | 8% |
|  |  |  |  | 42% | 38% | 42% | 37% | 33% | 28% | 33% | 29% |
| SlGGB2 |  |  |  |  | 51% | 30% | 9% | 7% | 4% | 5% | 6% |
|  |  |  |  |  | 72% | 51% | 35% | 31% | 30% | 27% | 29% |
| SlGGB1 |  |  |  |  |  | 28% | 8% | 7% | 4% | 5% | 7% |
|  |  |  |  |  |  | 46% | 31% | 27% | 23% | 25% | 29% |
| RGG2 |  |  |  |  |  |  | 8% | 8% | 4% | 6% | 6% |
|  |  |  |  |  |  |  | 37% | 33% | 30% | 37% | 31% |
| SlGGC1 |  |  |  |  |  |  |  | 44% | 20% | 26% | 32% |
|  |  |  |  |  |  |  |  | 70% | 51% | 53% | 51% |
| AGG3 |  |  |  |  |  |  |  |  | 20% | 25% | 27% |
|  |  |  |  |  |  |  |  |  | 53% | 53% | 50% |
| qPE9-1/DEP1 |  |  |  |  |  |  |  |  |  | 43% | 19% |
|  |  |  |  |  |  |  |  |  |  | 64% | 44% |
| GGC2 |  |  |  |  |  |  |  |  |  |  | 26% |
|  |  |  |  |  |  |  |  |  |  |  | 51% |

The top lines indicate the identity values detected using the whole region between any two proteins, while the bottom lines represent those detected using conserved GGL (G gamma-like) regions.

**Table S2** Major agronomic traits of NIP and *nrgg2-1*. Data are given as the means ± S.E. (n ≥ 15). Student’s *t-*test: **P* < 0.05; ***P* < 0.01.

| Traits | NIP | *nrgg2-1* |
| --- | --- | --- |
| Plant height (cm) | 94.35 ± 3.34 | 98.84 ± 2.27** |
| Panicle length (cm) | 21.67 ± 1.23 | 23.76 ± 2.07** |
| Panicle number per plant | 15.82 ± 4.75 | 15.21 ± 3.92 |
| Grain length (mm) | 7.46 ± 0.19 | 7.67 ± 0.19** |
| Grain width (mm) | 3.14 ± 0.14 | 3.12 ± 0.13 |
| Grain thickness (mm) | 2.14 ± 0.07 | 2.16 ± 0.06* |
| 1000-grain weight (g) | 24.42 ± 0.59 | 24.86 ± 0.44* |
| Grain number per panicle | 139.00 ± 13.82 | 136.64 ± 14.16 |
| Seed setting ratio (%) | 79.33 ± 4.68 | 76.63 ± 10.20 |
| Grain yield per plant (g) | 28.37 ± 5.32 | 28.47 ± 6.70 |
| Biomass yield per plant (g) | 62.35 ± 15.71 | 62.52 ± 14.00 |

**Table S3** Comparison of major agronomic traits between WYJ7 and the two overexpression lines of *RGG2*. WYJ7 is a *japonica* variety with the *qpe9-1* allele. WYJ7-OE1 and WYJ7-OE2 are two overexpression lines of *RGG2* in the WYJ7 background. Data are given as the means ± S.E. (n ≥ 16). Student’s *t-*tests: **P* < 0.05; ***P* < 0.01.

| Traits | WYJ7 | WYJ7-OE1 | WYJ7-OE2 |
| --- | --- | --- | --- |
| Plant height (cm) | 94.65 ± 3.59 | 81.70 ± 5.64** | 78.35 ± 3.14** |
| Panicle length (cm) | 16.93 ± 1.60 | 15.91 ± 0.93** | 15.02 ± 1.11** |
| Panicle number per plant | 8.95 ± 3.07 | 8.20 ± 3.52 | 7.40 ± 1.76 |
| Grain length (mm) | 7.40 ± 0.27 | 6.94 ± 0.17** | 6.84 ± 0.20** |
| Grain width (mm) | 3.27 ± 0.14 | 3.21 ± 0.20 | 3.26 ± 0.15 |
| Grain thickness (mm) | 2.32 ± 0.09 | 2.32 ± 0.10 | 2.33 ± 0.14 |
| 1000-grain weight (g) | 28.28 ± 0.42 | 24.32 ± 0.32** | 23.94 ± 0.50** |
| Grain number per panicle | 156.19 ± 28.49 | 143.50 ± 19.27 | 153.30 ± 22.57 |
| Setting percentage (%) | 91.13 ± 4.02 | 87.04 ± 7.96 | 94.06 ± 1.94 |
| Grain yield per plant (g) | 42.25 ± 12.33 | 23.84 ± 6.74** | 22.45 ± 9.47** |
| Biomass yield per plant (g) | 78.75 ± 32.42 | 44.90 ± 15.80** | 45.80 ± 18.81** |

**Table S4** Primers used in this study.

| Primer name | Sequence (5'-3') |
| --- | --- |
| RGG2-OE F | AAAGGATCCATGAGGGGGGAGGCGAACGGGGA |
| RGG2-OE R | AAAACTAGTCTAGGGAAAATCTGAGCCTTTGG |
| OsActin-qPCR F | GATGACCCAGATCATGTTTG |
| OsActin-qPCR R | GGGCGATGTAGGAAAGC |
| GGC2-qPCR F | GTGCAACTGCTTGTTATGCC |
| GGC2-qPCR R | GCTCGGTCTACAGCACGAT |
| GS3-qPCR F | CCGCGAGATCGGATTCC |
| GS3-qPCR R | CGTGGATCCCTTCGATTGA |
| qPE9-1-qPCR F | GGAGGAGGCGGTGGTGAT |
| qPE9-1-qPCR R | CACCGAAAAAGACGGCAAG |
| RGG1-qPCR F | AAACGGATAAGGTGTCAGCA |
| RGG1-qPCR R | TGCGGACCTTCAAACCA |
| RGG2-qPCR F | GCAGGATGAACTGAACGAGC |
| RGG2-qPCR R | GGATGCCCACCATTTGTTA |
| IPA1-qPCR F | GGATATGGTGCCAACACATACAG |
| IPA1-qPCR R | GACATGGCTGCAGCCTGGTTGTG |
| FZP-qPCR F | CTCCAGCATGTCGTCGTC |
| FZP-qPCR R | CACCACGCTGCTCAGGTA |
| EP2-qPCR F | AAGCTGCAAGGAGAACAGAAGG |
| EP2-qPCR R | AGGAGGAAACTTATGAGCGCAAC |
| PAP2-qPCR F | GCAGCTTGAGAGTCAAGTAGTC |
| PAP2-qPCR R | CCTTTCTCTTTAGGTCGCAGAG |
| SNB-qPCR F | GGTGGTTTCGATACAGCTCAT |
| SNB-qPCR R | GCTTCAAGTCGTCCTCATAGTC |
| LAX1-qPCR F | CATCAGATGATGCAGCAAGC |
| LAX1-qPCR R | GAAGACACAGCAAGGCAAAG |
| LAX2-qPCR F | AGGAGCTACCTACGGATCAA |
| LAX2-qPCR R | CCCTCACCTCTGATTCATCTTC |
| RGG2-GFP F | AAAAAGCTTATGAGGGGGGAGGCGAACGGGGAGG |
| RGG2-GFP R | AAAGGATCCTGGAAAAATCTGAGCCTTTGGATGCCCAC |
| RGG2 -BD F | AAAGAATTCATGAGGGGGGAGGCGAACGGGGAGG |
| RGG2 -BD R | AAAGGATCCCTAGGAAAAATCTGAGCCTTTGGATGCC |
| RGB-AD F | AAACCCGGGTATGGCGTCCGTGGCGGAGCTCA |
| RGB-AD R | AAAGGATCCTCAAACTATTTTCCGGTGTCCGCTGAA |
| RGG2 ΔC1-BD F | AAAGAATTCATGAGGGGGGAGGCGAACGGGGA |
| RGG2 ΔC1-BD R | AAAGGATCCCTCCCCCCACATCCGTCGGTCG |
| RGG2 ΔC2-BD R | AAAGGATCCCCTCGTTCAGTTCATCCTGCAGCGA |
| RGG2 ΔC3-BD R | AAAGGATCCCTGCAGCAGATGCCGGTTCCATGGT |
| RGG2 ΔC4-BD R | AAAGGATCCCGCATGCAGCAGATGCCGGTTCCAT |
| RGG2 ΔC5-BD R | AAAGGATCCCGGATTTTCCCTCTGTACTTGTGAT |
| RGG2 ΔC6-BD R | AAAGGATCCCGGTGACGGATTTTCCCTCTGTACTT |
| RGG2 ΔC7-BD R | AAAGGATCCCGGTGACAGGAAGAAGCGGGTCGGATT |
| RGG2 GGL-BD F | AAAGAATTCATGGGGGCGGCGATGAGGAGCGTG |
| RGG2-Pro-GUS F | AAAGAATTCCATAACAATTGGGCATGAATTAGGGTG |
| RGG2-Pro-GUS R | AAACCATGGTCATCTCCCCCTCACCACCACAACCA |
| RGG2-Cas9 F | GGCAGAGCTTGAAACCATGGAAC |
| RGG2-Cas9 R | AAACGTTCCATGGTTTCAAGCTC |
| RGG2-Cas9-seq F | AATCCTATTCGGTCCTTCTG |
| RGG2-Cas9-seq R | TACATTCGTCCATGTTCGTG |
| RGG2 gseq-1 F | GGTCCTACTACTTTCCCATCT |
| RGG2 gseq-1 R | GGCTTCTTATCTGAGGTTCG |
| RGG2 gseq-2 F | TTCCCGAAGTGCTAAAT |
| RGG2 gseq-2 R | AAGTCGTTGGTGGTGGT |
| RGG2 gseq-3 F | GCTGCCGTCCTCGCTAT |
| RGG2 gseq-3 R | CTCTCAAACTCGCAACCC |
| RGG2 gseq-4 F | TTATGAAAGCGTTGAACCA |
| RGG2 gseq-4 R | CCAGAAGGACCGAATAGGA |
| RGG2 gseq-5 F | CATACATTGGCAAGAAGAA |
| RGG2 gseq-5 R | CATCGCAATCTGAATGTTT |

**Table S5** Different haplotypes of *RGG2* in 132 rice germplasms.

| Germplasms | InDel1 | SNP1 | SNP2 | SNP3 | SNP4 | SNP5 | SNP6 | Class | Grain length (mm) | Grain width (mm) | Grain thickness (mm) | 1000-grain weight (g) | Haplotypes |
| --- | --- | --- | --- | --- | --- | --- | --- | --- | --- | --- | --- | --- | --- |
| NONABOKRA | - | A | T | A | A | A | T | *Indica* | 7.84 | 3.14 | 2.13 | 25.22 | Hap1 |
| C70 | - | A | T | A | A | A | T | *Indica* | 7.54 | 2.94 | 1.96 | 23.50 | Hap1 |
| Zaoxian 14 | - | A | T | A | A | A | T | *Indica* | 7.62 | 2.92 | 2.18 | 25.00 | Hap1 |
| R644 | - | A | T | A | A | A | T | *Indica* | 7.75 | 3.17 | 2.05 | 25.80 | Hap1 |
| Fengaizhan | - | A | T | A | A | A | T | *Indica* | 7.77 | 2.51 | 1.86 | 19.00 | Hap1 |
| RUSTIC::IRGC 117026-1 | - | A | T | A | A | A | T | *Indica* | 8.46 | 2.90 | 1.97 | 24.00 | Hap1 |
| VARY MALADY MENA::IRGC 51555-1 | - | A | T | A | A | A | T | *Indica* | 7.85 | 3.25 | 2.17 | 28.75 | Hap1 |
| PAI CHUEH CHIU LIU::IRGC 34259-1 | - | A | T | A | A | A | T | *Indica* | 7.53 | 3.32 | 2.28 | 21.20 | Hap1 |
| GENIT::IRGC 3272-1 | - | A | T | A | A | A | T | *Indica* | 8.12 | 2.87 | 2.21 | 26.33 | Hap1 |
| IR 57920-AC 25-2-B::C1 | - | A | T | A | A | A | T | *Indica* | 7.69 | 3.21 | 2.22 | 27.40 | Hap1 |
| Qingsiai16B | - | A | T | A | A | A | T | *Indica* | 7.54 | 3.15 | 2.25 | 26.80 | Hap1 |
| Keten Nangka/02428 | - | A | T | A | A | A | T | *Indica* | 9.70 | 2.77 | 2.14 | 28.93 | Hap1 |
| Huangsiguizhan | - | A | T | A | A | A | T | *Indica* | 9.51 | 2.65 | 2.06 | 26.40 | Hap1 |
| Xiangzaoxian 7 | - | A | T | A | A | A | T | *Indica* | 9.91 | 2.72 | 2.16 | 28.00 | Hap1 |
| IRRI 146::G1 | - | A | T | A | A | A | T | *Indica* | 7.98 | 2.93 | 2.17 | 24.60 | Hap1 |
| TAIPEI 167::IRGC 65371-1 | - | A | T | A | A | A | T | *Indica* | 8.37 | 3.14 | 2.19 | 27.50 | Hap1 |
| Zhenshan 97 | - | A | C | G | G | G | T | *Indica* | 7.99 | 3.06 | 1.86 | 20.21 | Hap2 |
| UPR 191-66 | - | A | C | G | G | G | T | *Indica* | 9.06 | 2.56 | 2.08 | 25.93 | Hap2 |
| Chenhui 448 | - | A | C | G | G | G | T | *Indica* | 7.60 | 3.10 | 2.07 | 24.70 | Hap2 |
| CAUVERY::IRGC 45255-1 | - | A | C | G | G | G | T | *Indica* | 10.50 | 2.54 | 2.12 | 29.15 | Hap2 |
| IRGA 318-11-9-2A::IRGC 117340-1 | - | A | C | G | G | G | T | *Indica* | 7.86 | 2.94 | 1.97 | 22.50 | Hap2 |
| PALEPYU::IRGC 33549-1 | - | A | C | G | G | G | T | *Indica* | 9.10 | 2.90 | 2.02 | 26.65 | Hap2 |
| IRI 339::IRGC 46956-1 | - | A | C | G | G | G | T | *Indica* | 8.68 | 2.51 | 2.01 | 22.50 | Hap2 |
| DALSUNG 41::IRGC 79385-1 | - | A | C | G | G | G | T | *Indica* | 10.22 | 2.54 | 2.04 | 28.95 | Hap2 |
| UPRH 233::IRGC 61667-1 | - | A | C | G | G | G | T | *Indica* | 7.84 | 2.92 | 2.00 | 22.50 | Hap2 |
| IR 57920-AC 25-2-B::C1 | - | A | C | G | G | G | T | *Indica* | 8.61 | 3.01 | 2.08 | 25.40 | Hap2 |
| TKM 9 | - | A | C | G | G | G | T | *Indica* | 8.12 | 2.97 | 2.13 | 25.17 | Hap2 |
| Huangguangyouzhan | - | A | C | G | G | G | T | *Indica* | 9.85 | 2.36 | 2.04 | 22.35 | Hap2 |
| Yuehesimiao | - | A | C | G | G | G | T | *Indica* | 9.87 | 2.14 | 1.90 | 17.40 | Hap2 |
| Huanghuazhan | - | A | C | G | G | G | T | *Indica* | 9.40 | 2.42 | 1.93 | 21.60 | Hap2 |
| Guangluai 4 | - | A | C | G | G | G | T | *Indica* | 8.71 | 3.01 | 2.16 | 25.35 | Hap2 |
| 9311 | - | A | C | G | G | G | T | *Indica* | 9.34 | 2.35 | 1.93 | 23.87 | Hap2 |
| 9311K | - | A | C | G | G | G | T | *Indica* | 9.32 | 2.42 | 2.05 | 25.70 | Hap2 |
| Wushansimiao | - | A | C | G | G | G | T | *Indica* | 9.51 | 2.21 | 1.92 | 19.90 | Hap2 |
| TGMS29 | - | A | T | G | G | A | T | *Indica* | 8.17 | 2.70 | 2.07 | 20.67 | Hap3 |
| CHANDINA::IRGC 36420-1 | - | A | T | G | G | A | T | *Indica* | 7.93 | 3.05 | 2.04 | 26.90 | Hap3 |
| ROXANI::GERVEX 1686-C1 | - | A | T | G | G | A | T | *Indica* | 8.29 | 2.73 | 2.12 | 21.75 | Hap3 |
| RACE PERUMAL::IRGC 55347-1 | - | G | T | G | G | A | A | *Indica* | 8.99 | 2.99 | 2.03 | 25.50 | Hap4 |
| YA NONG ZAO 4::IRGC 63908-1 | - | A | C | G | G | G | A | *Indica* | 8.55 | 2.76 | 1.97 | 22.47 | Hap5 |
| GIZA 178::GERVEX 1681-C1 | - | A | C | G | G | G | A | *Indica* | 7.93 | 2.83 | 2.08 | 23.40 | Hap5 |
| Baikehanhe | - | A | C | G | G | G | A | *Indica* | 9.24 | 2.86 | 1.95 | 26.37 | Hap5 |
| Nipponbare | - | A | T | A | A | A | T | *Japonica* | 7.56 | 3.10 | 2.11 | 24.42 | Hap1 |
| 68-2::IRGC 14546-1 | - | A | T | A | A | A | T | *Japonica* | 8.13 | 3.19 | 2.06 | 26.60 | Hap1 |
| YONG AN HUK::IRGC 19891-1 | - | A | T | A | A | A | T | *Japonica* | 8.07 | 2.94 | 2.10 | 26.50 | Hap1 |
| YRM 6-2::GERVEX 1508-C1 | - | A | T | A | A | A | T | *Japonica* | 7.81 | 2.99 | 1.92 | 22.92 | Hap1 |
| TIMICH 108::GERVEX 1325-C1 | - | A | T | A | A | A | T | *Japonica* | 8.32 | 3.11 | 2.22 | 26.53 | Hap1 |
| THAIPERLA::GERVEX 696-C1 | - | A | T | A | A | A | T | *Japonica* | 8.20 | 3.01 | 2.18 | 27.50 | Hap1 |
| SUPER::GERVEX 1304-C1 | - | A | T | A | A | A | T | *Japonica* | 8.79 | 3.03 | 2.26 | 26.60 | Hap1 |
| SR 113::GERVEX 553-C1 | - | A | T | A | A | A | T | *Japonica* | 7.28 | 3.01 | 2.21 | 22.70 | Hap1 |
| SMERALDO::GERVEX 138-C1 | - | A | T | A | A | A | T | *Japonica* | 8.52 | 3.15 | 2.26 | 28.60 | Hap1 |
| SALOIO::GERVEX 1259-C1 | - | A | T | A | A | A | T | *Japonica* | 7.85 | 2.90 | 1.94 | 23.20 | Hap1 |
| RUBINO::GERVEX 80-C1 | - | A | T | A | A | A | T | *Japonica* | 8.43 | 2.59 | 2.07 | 22.93 | Hap1 |
| RODINA::GERVEX 1234-C1 | - | A | T | A | A | A | T | *Japonica* | 8.41 | 2.80 | 2.19 | 24.83 | Hap1 |
| LOMELLINO::GERVEX 83-C1 | - | A | T | A | A | A | T | *Japonica* | 7.11 | 3.85 | 2.69 | 32.33 | Hap1 |
| L 205::GERVEX 1659-C1 | - | A | T | A | A | A | T | *Japonica* | 7.54 | 3.42 | 2.32 | 27.10 | Hap1 |
| IRAT 335::C1 | - | A | T | A | A | A | T | *Japonica* | 7.19 | 3.25 | 2.26 | 29.00 | Hap1 |
| IBO 400::GERVEX 943-C1 | - | A | T | A | A | A | T | *Japonica* | 8.44 | 3.03 | 1.99 | 26.50 | Hap1 |
| HARRA::GERVEX 501-C1 | - | A | T | A | A | A | T | *Japonica* | 8.63 | 3.05 | 2.03 | 24.90 | Hap1 |
| GOLFO::GERVEX 118-C1 | - | A | T | A | A | A | T | *Japonica* | 7.67 | 3.10 | 2.21 | 26.85 | Hap1 |
| CIGALON::GERVEX 1514-C1 | - | A | T | A | A | A | T | *Japonica* | 6.92 | 3.12 | 2.11 | 25.33 | Hap1 |
| CAPATAZ::GERVEX 521-C1 | - | A | T | A | A | A | T | *Japonica* | 7.89 | 3.08 | 2.24 | 27.30 | Hap1 |
| ANSEATICO::GERVEX 67-C1 | - | A | T | A | A | A | T | *Japonica* | 9.31 | 2.64 | 2.06 | 25.87 | Hap1 |
| Gongchengxiang | - | A | T | A | A | A | T | *Japonica* | 10.65 | 2.63 | 2.10 | 30.90 | Hap1 |
| Qiuguang tengxi104 | - | A | T | A | A | A | T | *Japonica* | 8.43 | 3.00 | 2.16 | 29.22 | Hap1 |
| KINUGASAWASE::IRGC 2609-1 | - | A | T | A | A | A | T | *Japonica* | 9.49 | 3.10 | 2.21 | 32.44 | Hap1 |
| Lian 16 | - | A | T | A | A | A | T | *Japonica* | 7.10 | 2.94 | 2.27 | 24.08 | Hap1 |
| 2845 | - | A | T | A | A | A | T | *Japonica* | 6.76 | 3.06 | 2.18 | 24.78 | Hap1 |
| Lian 15 | - | A | T | A | A | A | T | *Japonica* | 6.82 | 3.08 | 2.41 | 24.42 | Hap1 |
| Shengdao 15 | - | A | T | A | A | A | T | *Japonica* | 7.29 | 3.13 | 2.48 | 27.50 | Hap1 |
| Shengdao 13 | - | A | T | A | A | A | T | *Japonica* | 7.00 | 2.97 | 2.34 | 23.60 | Hap1 |
| Shengdao 22 | - | A | T | A | A | A | T | *Japonica* | 7.28 | 2.98 | 2.31 | 23.60 | Hap1 |
| Shengdao 14 | - | A | T | A | A | A | T | *Japonica* | 7.00 | 3.03 | 2.40 | 25.60 | Hap1 |
| Shengdao 16 | - | A | T | A | A | A | T | *Japonica* | 7.41 | 3.18 | 2.46 | 27.80 | Hap1 |
| Shengdao 17 | - | A | T | A | A | A | T | *Japonica* | 7.34 | 2.95 | 2.31 | 24.75 | Hap1 |
| Shengdao 18 | - | A | T | A | A | A | T | *Japonica* | 7.14 | 2.96 | 2.29 | 24.15 | Hap1 |
| Shengdao 19 | - | A | T | A | A | A | T | *Japonica* | 7.08 | 3.09 | 2.43 | 25.35 | Hap1 |
| Shengdao 20 | - | A | T | A | A | A | T | *Japonica* | 7.66 | 3.29 | 2.49 | 26.40 | Hap1 |
| Shengdao 53 | - | A | T | A | A | A | T | *Japonica* | 7.17 | 2.85 | 2.11 | 23.00 | Hap1 |
| Shengdao 25 | - | A | T | A | A | A | T | *Japonica* | 7.51 | 2.98 | 2.39 | 25.85 | Hap1 |
| Shengdao 2572 | - | A | T | A | A | A | T | *Japonica* | 7.63 | 3.18 | 2.33 | 27.13 | Hap1 |
| Shengdao 24 | - | A | T | A | A | A | T | *Japonica* | 7.24 | 3.02 | 2.27 | 24.77 | Hap1 |
| Runnong 11 | - | A | T | A | A | A | T | *Japonica* | 7.61 | 3.26 | 2.41 | 25.90 | Hap1 |
| Lindao 10 | - | A | T | A | A | A | T | *Japonica* | 7.10 | 2.95 | 2.07 | 23.27 | Hap1 |
| Lindao 20 | - | A | T | A | A | A | T | *Japonica* | 7.21 | 3.02 | 2.43 | 28.27 | Hap1 |
| Shengdao 72 | - | A | T | A | A | A | T | *Japonica* | 7.54 | 3.07 | 2.33 | 29.60 | Hap1 |
| Lindao 16 | - | A | T | A | A | A | T | *Japonica* | 7.01 | 3.29 | 2.61 | 31.70 | Hap1 |
| Shengdao 23 | - | A | T | A | A | A | T | *Japonica* | 7.13 | 3.00 | 2.35 | 26.30 | Hap1 |
| Yangguang 800 | - | A | T | A | A | A | T | *Japonica* | 6.97 | 3.14 | 2.30 | 29.10 | Hap1 |
| Zhehujing 25 | - | A | T | A | A | A | T | *Japonica* | 7.16 | 2.90 | 2.20 | 22.03 | Hap1 |
| Zhejing 96 | - | A | T | A | A | A | T | *Japonica* | 7.66 | 2.93 | 2.20 | 24.93 | Hap1 |
| Zhejing 99 | - | A | T | A | A | A | T | *Japonica* | 7.46 | 3.05 | 2.25 | 23.54 | Hap1 |
| Zhejing 59 | - | A | T | A | A | A | T | *Japonica* | 7.55 | 2.92 | 2.22 | 25.33 | Hap1 |
| Songjing 19 | - | A | T | A | A | A | T | *Japonica* | 9.03 | 2.80 | 2.12 | 27.63 | Hap1 |
| Songjing 3 | - | A | T | A | A | A | T | *Japonica* | 6.77 | 3.13 | 2.29 | 24.17 | Hap1 |
| Shennong15256 | - | A | T | A | A | A | T | *Japonica* | 7.60 | 3.06 | 2.24 | 25.83 | Hap1 |
| Longjing 31 | - | A | T | A | A | A | T | *Japonica* | 7.02 | 3.12 | 2.31 | 25.40 | Hap1 |
| Liaojing 401 | - | A | T | A | A | A | T | *Japonica* | 7.58 | 2.81 | 2.14 | 23.67 | Hap1 |
| Longdao 18 | - | A | T | A | A | A | T | *Japonica* | 7.99 | 2.75 | 2.05 | 25.07 | Hap1 |
| Ji 14-115 | - | A | T | A | A | A | T | *Japonica* | 7.24 | 2.93 | 2.13 | 23.90 | Hap1 |
| Yueguang | - | A | T | A | A | A | T | *Japonica* | 7.09 | 3.24 | 2.32 | 26.77 | Hap1 |
| Fengjin | - | A | T | A | A | A | T | *Japonica* | 7.31 | 3.25 | 2.30 | 27.17 | Hap1 |
| Pinyi | - | A | T | A | A | A | T | *Japonica* | 7.32 | 2.76 | 1.98 | 24.97 | Hap1 |
| Yunlangxiang | - | A | T | A | A | A | T | *Japonica* | 6.56 | 2.95 | 2.13 | 19.87 | Hap1 |
| Qiutianxiaoding | - | A | T | A | A | A | T | *Japonica* | 7.22 | 3.03 | 2.22 | 24.33 | Hap1 |
| Daohuaxiang 2 | - | A | T | A | A | A | T | *Japonica* | 8.76 | 2.59 | 2.10 | 24.93 | Hap1 |
| Suken 118 | - | A | T | A | A | A | T | *Japonica* | 7.45 | 3.28 | 2.33 | 25.37 | Hap1 |
| Wuyunjing 7 | - | A | T | A | A | A | T | *Japonica* | 7.40 | 3.27 | 2.32 | 28.28 | Hap1 |
| Yandao 8 | - | A | T | A | A | A | T | *Japonica* | 7.16 | 3.10 | 2.34 | 23.53 | Hap1 |
| Lianjing 63 | - | A | T | A | A | A | T | *Japonica* | 7.73 | 3.11 | 2.28 | 26.07 | Hap1 |
| Dongjing | - | A | T | A | A | A | T | *Japonica* | 7.28 | 3.11 | 2.38 | 25.53 | Hap1 |
| Hwaying | - | A | T | A | A | A | T | *Japonica* | 7.30 | 3.09 | 2.27 | 23.53 | Hap1 |
| SKY BONNET::IRGC 66759-1 | - | A | C | G | G | G | T | *Japonica* | 8.61 | 2.80 | 1.95 | 22.73 | Hap2 |
| Shennong 265 | - | A | C | G | G | G | T | *Japonica* | 9.80 | 2.64 | 2.10 | 28.00 | Hap2 |
| KALIN::IRGC 77312-1 | - | A | C | G | G | G | T | *Japonica* | 8.80 | 2.77 | 2.06 | 24.78 | Hap2 |
| HOKURIKU 52::IRGC 72491-1 | - | A | C | G | G | G | T | *Japonica* | 9.41 | 2.42 | 1.88 | 22.93 | Hap2 |
| BLUE BELLE::IRGC 51125-1 | - | A | C | G | G | G | T | *Japonica* | 7.52 | 3.06 | 2.07 | 25.25 | Hap2 |
| SHSS 53::GERVEX 550-C1 | - | A | C | G | G | G | T | *Japonica* | 8.70 | 2.65 | 2.17 | 25.67 | Hap2 |
| MELAS::GERVEX 1684-C1 | - | A | C | G | G | G | T | *Japonica* | 9.66 | 2.68 | 2.04 | 27.92 | Hap2 |
| WIR 2091::IRGC 57536-1 | - | A | C | G | G | G | T | *Japonica* | 7.35 | 3.01 | 2.12 | 24.75 | Hap2 |
| ITALPATNA 48::GERVEX 60-C1-G1 | - | A | C | G | G | G | T | *Japonica* | 9.66 | 2.57 | 2.02 | 26.20 | Hap2 |
| GHIBLI::GERVEX 187-C1 | - | A | T | G | G | A | T | *Japonica* | 9.96 | 2.65 | 2.12 | 29.17 | Hap3 |
| Wanshi | - | A | T | G | G | A | T | *Japonica* | 7.74 | 3.05 | 2.19 | 25.10 | Hap3 |
| S 201::IRGC 55230-1 | - | G | T | G | G | A | A | *Japonica* | 8.97 | 2.59 | 2.12 | 24.00 | Hap4 |
| JAPONES BALILLA::IRGC 5785-1 | - | G | T | G | G | A | A | *Japonica* | 9.44 | 2.66 | 2.03 | 27.00 | Hap4 |
| SAKHA 103::GERVEX 1688-C1 | - | G | T | G | G | A | A | *Japonica* | 8.72 | 2.87 | 2.18 | 25.89 | Hap4 |
| SAKHA 102::GERVEX 1687-C1 | - | G | T | G | G | A | A | *Japonica* | 8.55 | 2.56 | 1.95 | 21.75 | Hap4 |
| IR 68704-145-1-1-B::C1 | - | G | T | G | G | A | A | *Japonica* | 9.14 | 2.64 | 1.98 | 23.20 | Hap4 |
| GRAAL::GERVEX 1682-C1 | - | G | T | G | G | A | A | *Japonica* | 7.51 | 2.68 | 1.91 | 19.10 | Hap4 |
| 80050YR72136-43 | - | G | T | G | G | A | A | *Japonica* | 9.60 | 2.87 | 2.12 | 28.80 | Hap4 |
| LOTO::GERVEX 104-C1 | - | A | C | G | G | G | A | *Japonica* | 8.69 | 2.99 | 1.97 | 25.17 | Hap5 |
| Suyunuo | GGA | A | T | A | A | A | T | *Japonica* | 10.01 | 3.44 | 2.20 | 36.83 | Hap6 |
| Dalijing | GGA | A | T | A | A | A | T | *Japonica* | 9.99 | 3.66 | 2.31 | 39.37 | Hap6 |
